# Integrating theory and machine learning to reveal determinants of plasmid copy number

**DOI:** 10.1038/s41467-026-72303-0

**Published:** 2026-04-22

**Authors:** Iqra Shahzadi, Wenzhi Xue, Hasan Ubaid Ullah, Rohan Maddamsetti, Lingchong You, Teng Wang

**Affiliations:** 1 State Key Laboratory of Quantitative Synthetic Biology, Shenzhen Institute of Synthetic Biology, Shenzhen Institutes of Advanced Technology, Chinese Academy of Sciences, Shenzhen, China; 2 University of Chinese Academy of Sciences, Beijing, China; 3 Department of Biochemistry and Microbiology, Rutgers University, New Brunswick, NJ USA; 4 Center for Quantitative Biodesign, Duke University, Durham, NC USA; 5 Department of Biomedical Engineering, Duke University, Durham, NC USA; 6 Department of Molecular Genetics and Microbiology, Duke University School of Medicine, Durham, NC USA

**Keywords:** Bacterial genetics, Microbial ecology, Bacterial evolution

## Abstract

Plasmids are extrachromosomal mobile genetic elements whose copy numbers (PCNs) critically influence microbial evolution, antibiotic resistance and pathogenicity. Despite their importance and immense diversity, the ecological, evolutionary and molecular factors determining PCN remain poorly understood. Here, we present a theoretical model to explain the empirical power-law relationship between plasmid size and copy number, one of the fundamental quantitative principles governing PCN control. However, this relationship alone has limited predictive power. To improve PCN prediction, we introduce a data-driven approach incorporating diverse features. Trained and tested on 11,051 plasmids, our machine learning model achieves significantly enhanced accuracy, with plasmid-encoded protein domains emerging as key predictors. Applying this framework, we conduct a large-scale analysis of PCN distributions across hundreds of thousands of metagenomic plasmids (IMG/PR database) and tens of thousands of clinical isolates, revealing putative niche specific taxonomic PCN hotspots and hypothesis-generating ecological trends. These results provide valuable insights into plasmid ecology, antibiotic resistance genes (ARGs) surveillance and shed lights on the gut plasmidome, a “dark matter” in human microbiome.

## Abstract (web summary)

Authors use machine learning to predict plasmid copy numbers across diverse ecosystems. They identify protein domains as one of the key determinants and reveal ecological trends relevant to antibiotic resistance surveillance.

## Introduction

Plasmids are extrachromosomal mobile genetic elements that play critical roles in microbial genomics, ecology and evolution^1^. Plasmids typically exist in multiple copies per cell, with copy numbers (PCNs) spanning several orders of magnitude^2,3^. PCN holds significance in multiple scenarios, from the environment, health, to engineering. Multicopy plasmids can potentiate bacterial evolution, promoting the emergence and dissemination of antibiotic resistance^4–6^. Many virulence factors are plasmid-encoded, with PCN variation playing a pivotal role in the pathogenesis of microbes such as *Yersinia pestis*, the plague bacterium^7–9^. In genetic engineering, plasmids serve as invaluable tools, where PCN directly determines the gene dosage, expression level, metabolic burden and circuit stability^10–13^.

Natural environments harbor a vast diversity of plasmids, evidenced by repositories like the global IMG/PR database, which currently houses nearly 700,000 plasmid sequences from different ecosystems, from the human gut to the ocean^14^. By contrast, experimental measurements of PCN (e.g., via qPCR or Southern blot) remain labor-intensive and low-throughput^15^. While high-throughput sequencing enables PCN estimation through coverage ratio analysis (plasmid versus chromosomal reads), current pipelines have only characterized PCNs for about 12,000 plasmids—a small fraction of natural plasmid diversity—leaving the copy numbers of most plasmids unknown^2,3^.

This striking disparity highlights the necessity of computational PCN predictions. Large-scale PCN predictions will help deduce the quantitative principles and ecological patterns governing copy number variations. Such predictive capability is particularly valuable in environments like the human gut, where the uncultivability of many microbial organisms makes direct PCN measurement experimentally challenging^16^. Moreover, given the growing antibiotic resistance crisis, reliable PCN prediction would enable precise assessment of plasmid-borne resistance gene dosage, thereby improving our ability to evaluate and control dissemination risk^17^. Additionally, accurate PCN prediction would support the rational design of synthetic gene circuits, facilitating the development of predictable synthetic biology systems^18^.

Previous studies identified an inverse power-law relationship between plasmid length and copy number, one of the fundamental rules governing PCN^2,3^. However, as explicitly noted by these studies, the underlying molecular mechanisms and evolutionary origins of such scaling remain unclear. Here, we first developed a theoretical model to explain this empirical relationship, confirming plasmid size as an important PCN predictor. However, this relationship alone has limited predictive power. To overcome this limitation, we designed a machine learning framework incorporating diverse plasmid features, which significantly improved prediction accuracy and highlighted the role of plasmid-encoded protein domains in shaping PCN. Applying this model to clinical plasmids revealed the quantitative interplay between PCN and antibiotic resistance gene (ARG) dosage. We further applied this framework to the IMG/PR dataset, which enabled the large-scale PCN predictions for metagenomic plasmids, revealing putative ecosystem-specific PCN trends, particularly in the human gut microbiome, where organism uncultivability precludes a lot of direct measurements. Combining theoretical modeling and data-driven approaches, this work provides mechanistic insights for empirically observed scaling laws and offers a framework for robust PCN prediction across diverse ecosystems, with broad implications for microbial ecology, evolution, and human health.

## Results

### A simple theory explains how PCN correlates with plasmid size

To uncover the quantitative principles governing PCN regulation, we first analyzed a comprehensive PCN dataset developed in a previous study^3^ (“Methods”). This dataset, encompassing 11,338 prokaryotic plasmids, was built upon 4317 plasmid-carrying genomes whose short-read sequencing data were provided in the NCBI Sequence Read Archive (SRA)^19^. For each plasmid, PCN was estimated by calculating its mean sequencing coverage relative to that of its host chromosome, assuming that the abundances of reads mapping to the chromosome or plasmids reflected their physical DNA amounts in the host cell. Note that these PCN estimates represent relative copy numbers (plasmid-to-chromosome ratios), not necessarily absolute plasmid counts per cell^3^. PCN values may fall below one if the cell contains multiple chromosome copies or if plasmids are absent in some cells of the sequenced population^20–23^.

The majority (99.77%) of plasmids in the dataset are from bacteria, while the rest are from archaea. *Escherichia* accounts for the largest proportion (~20%) of plasmids, followed by *Klebsiella* (~18%) and Salmonella (~5%). Plasmid length varied significantly, ranging from 1 kb to 2.59 Mb, with a mean of 94 kb and a median of 55.8 kb. While most plasmids in the dataset come from natural environments, 0.2% are engineered (constructed or modified in laboratories, see “Methods”). Ecologically, the dataset covers diverse microbial ecosystems, with the human-associated environment contributing the largest proportion of plasmids (37.7%), followed by livestock (7.37%).

**Fig. 1:**
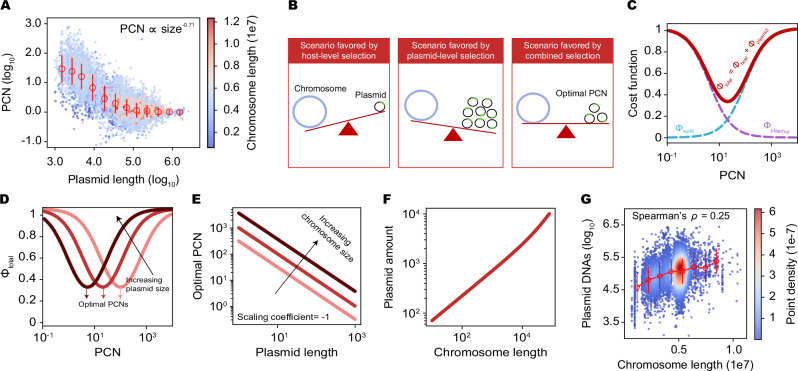
Multi-level selections explain the power-law relationship between plasmid size and plasmid copy number (PCN). **A** The inverse power-law relationship between plasmid size and PCN, with host chromosome length indicated by color gradient. Larger plasmids generally have lower copy numbers, reflecting an evolutionary size-PCN tradeoff (*n* = 11,051 plasmids). The x-axis was partitioned into multiple logarithmically spaced size bins. The mean ± S.D. of log10-transformed PCN values within each bin is shown. **B** The plasmid size-PCN tradeoff arises from the interplay between two selective forces. Host-level selection favors plasmids with minimal metabolic burden (left panel), while plasmid-level selection favors those with maximized self-replication and transfer (middle panel). The optimal PCN emerges from the interplay of these two opposing forces (right panel). **C** Theoretical curves showing the cost functions for host-level selection (${\phi }_{h{ost}}$, blue dashed line) and plasmid-level selection (${\phi }_{{plasmid}}$, purple dashed line). Selection at each level operates by minimizing its respective cost function. Because these objectives are inherently conflicting, the optimal PCN emerges as the value that minimizes the combined cost (${\phi }_{t{otal}}={\phi }_{h{ost}}+{\phi }_{p{lasmid}}$, red solid line). ${\phi }_{h{ost}}$ was formulated as ${\phi }_{h{ost}}=\frac{h\theta P}{K+C+h\theta P}$, where $C$, $P$ and $\theta$ represent chromosome size, plasmid size and PCN, respectively. $K$ is a constant characterizing how the total amount of intracellular resources changes with genome size. $h$ is a constant describing the relative advantage of plasmid genes in resource competition. ${\phi }_{p{lasmid}}$ was formulated as ${\phi }_{p{lasmid}}=\frac{C}{C+h\theta P}.$ Here, the theoretical curves were generated with $K=5\times {10}^{4}$, $C=2000$, $P=50$ and $h=10$. Varying parameters did not change the shapes of the curves. **D** The relationships between ${\phi }_{t{otal}}$ and PCN under different plasmid sizes. There are different plasmid sizes ($P$ = 10, 50 and 200 from right to left) that were tested. Other parameters are $K=5\times {10}^{4}$, $C=2000$ and $h=10$. **E** Theoretically predicted plasmid size-PCN tradeoffs under three different chromosome sizes. Here, the scaling exponent between plasmid size and PCN equals −1. Three different chromosome sizes ($C$ = $2\times {10}^{2}$, $2\times {10}^{3}$ and $2\times {10}^{4}$ from left to right) were tested with $K=5\times {10}^{4}$ and $h=10$. **F** Theoretically predicted positive correlation between chromosome length and plasmid DNA amount. Here, the plasmid DNA amount was calculated as plasmid size multiplied by its PCN. Other parameters are $K=5\times {10}^{4}$ and $h=10$. **G** Observed positive correlation (Spearman’s *ρ* = 0.25) between chromosome length and plasmid DNA amount, with point density represented by color shading. Each data point represents an individual plasmid (*n* = 11,051). The mean ± S.D. of log10-transformed PCN values within each bin is shown as red circles.

This dataset revealed an inverse power law correlation between plasmid length and PCN (Fig. 1A), characterized by a scaling exponent of −0.71. To statistically validate this relationship, we performed two complementary analyses. Bootstrap resampling (*n* = 1000 iterations) confirmed the scaling exponent is highly stable, with a narrow 95% confidence interval being –0.719 to –0.696 (Supplementary Fig. S1A). Formal model comparison using the Akaike Information Criterion (AIC) decisively favored the power-law model over alternative functional forms, including linear, exponential, and log-linear models (ΔAIC > 9000 relative to the next-best alternative, Supplementary Fig. S1B). This relationship suggests a tradeoff between plasmid length and copy number, where larger plasmids generally maintain lower copy number, a pattern also reported in previous studies^2,3^. Yet, the quantitative basis of this power-law relationship remains unclear.

From first principles, the persistence of a plasmid in microbial genomes was shaped by two levels of selective forces: host-level selection and plasmid-level selection within a host (Fig. 1B)^24,25^. Host-level selection tends to eliminate plasmids that impose a high metabolic burden. This occurs because plasmid gene expression consumes energy and resources in the host cell, reducing host fitness and causing plasmid-bearing cells to be competitively excluded by plasmid-free ones. However, even burdensome plasmids may persist by exploiting strategies such as highly efficient replication or horizontal transfer^26,27^. These plasmid-centric forces, collectively referred to as plasmid-level selection^24,25^, encompass all mechanisms that increase plasmid persistence at the host’s expense, including plasmid replication rate, transfer efficiency, and reduced segregation error. Long-term plasmid stability in prokaryotic genomes thus emerges from the interplay of these opposing forces.

To explain the quantitative tradeoff between plasmid size and PCN, we developed a mathematical model in which host- and plasmid-level forces are formalized as outcomes of intracellular resource competition between chromosomes and plasmids^24^ (see Supplementary Information for details). In this framework, plasmid competitiveness scales with its total DNA content (plasmid size × PCN). We then defined the cost functions for host-level (${\phi }_{{host}}$) and plasmid-level (${\phi }_{{plasmid}}$) forces, which could be derived analytically as Hill functions of chromosome size and plasmid DNA content (Fig. 1C). Selection at each level operates by minimizing its respective cost function. Because these objectives are inherently conflicting, the optimal PCN emerges as the value that minimizes the combined cost function (${\phi }_{{total}}$), defined as the sum of ${\phi }_{{host}}$ and ${\phi }_{{plasmid}}$ (Fig. 1C). In this way, plasmid evolution is treated as an optimization problem. Owing to the model’s simplicity, the optimal PCN can be solved analytically as a function of both chromosome and plasmid size (Fig. 1D).

The theory successfully predicts the power-law relationship between plasmid size and PCN, albeit with a scaling exponent of −1 (Fig. 1E). In this theory, the exponent of −1 arises under simplifying mean-field assumptions, including uniform gene density across plasmid sizes, size-independent regulatory costs, functional equivalence of genes in terms of competitiveness, and a constant average metabolic burden per plasmid copy imposed on the host. Within this idealized framework, copy number scales inversely with plasmid size because each additional copy contributes proportionally to host cost. The primary aim of this analytical model is to demonstrate the fundamental origin of inverse scaling rather than to precisely fix its exponent.

Therefore, the discrepancy between the predicted and observed scaling exponents likely reflects the model’s intentional simplifications. For instance, the model didn’t account for the functional heterogeneity of chromosome or plasmid-encoded genes. Additionally, it overlooked specific gene conflicts between plasmids and chromosomes, an important source of plasmid fitness burdens^28^. Nevertheless, the model still predicts a positive correlation between plasmid DNA content and chromosome size (Fig. 1F), which was confirmed by PCN dataset analysis (Spearman’s $\rho$ = 0.25; Fig. 1G, Supplementary Fig. S1C)^2^. Together, these findings provide a mechanistic basis for understanding the copy number control of prokaryotic plasmids.

While our theory provides a plausible explanation for the tradeoff between PCN and plasmid size, its quantitative predictive power remains limited (Fig. 1G). The power law fit is also inadequate (*R*² ~ 0.63, Supplementary Fig. S1D), as it ignores other factors influencing copy number regulation, especially plasmid-encoded functions. This gap underscores the necessity for a data-driven approach to model PCN that considers genetic features beyond plasmid length.

### PCN prediction through a multi-feature machine learning framework

To improve prediction accuracy, we developed a random forest regression model trained on diverse genomic and plasmid-level features, with a strategic emphasis on plasmid-encoded protein domains. This domain-centric approach addresses a key limitation in plasmid biology: many plasmid-encoded proteins lack functional annotations in public databases and are simply labeled as ‘hypothetical’^29^. Traditional analyses relying on annotated proteins risk overlooking these uncharacterized elements^30^, biasing predictions toward well-studied systems and limiting their applicability in many real-world scenarios like metagenomes, where functional annotations can be sparse. By contrast, protein domains, detectable even in unannotated sequences, represent evolutionarily conserved functional units^31^. This framework ensures that our model remains robust across diverse datasets, including poorly annotated plasmids from clinical or environmental isolates^14^.

**Fig. 2:**
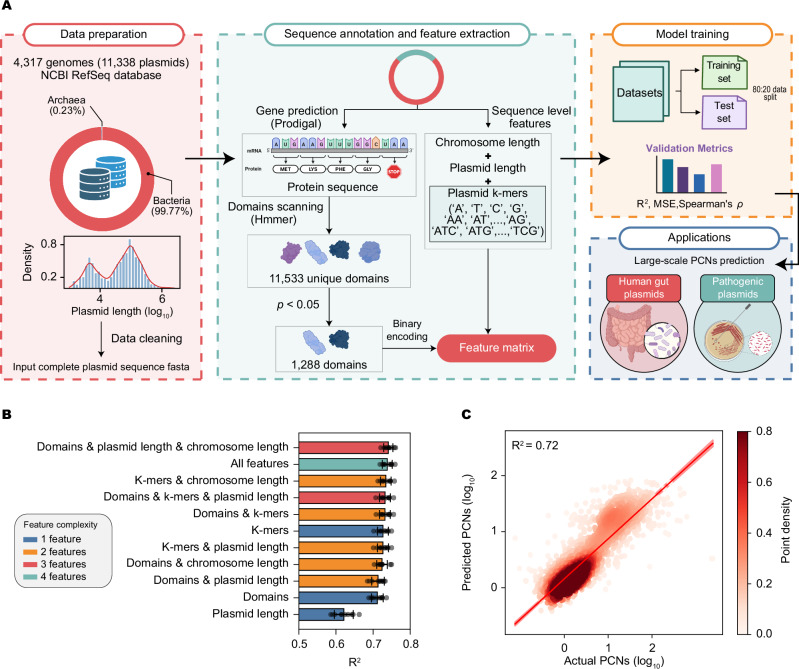
A machine learning framework that predicts plasmid copy number (PCN) using sequence-derived features. **A** The computational pipeline. We retrieved 11,338 plasmid sequences from 4317 genomes (99.77% bacterial, 0.23% archaeal). Protein domains were annotated using HMMER (Pfam), with 1288 domains showing significant correlation with PCN. Features (plasmid length, chromosome length, *k*-mers, plasmid encoded domains) were combined into a matrix. The dataset was split at a 4:1 ratio for training and test, and performance was evaluated with R², MSE, and Spearman’s *ρ*. The figure was created in BioRender. Wang, T. (2026) https://BioRender.com/wcaten9. **B** Multi-feature models (domains, *k*-mers, replicon lengths) achieved higher *R²* values, outperforming single-feature models. Error bars represent the standard deviations around the mean across 10 replicates. Source data are provided as a Source Data file. **C** The correlation between actual PCN values and those predicted by the model using all features. Point density is represented by red shading (darker indicates higher density).

We first used Prodigal to identify protein-coding sequences (CDSs) of all plasmids^32^. Domain annotation of these CDSs against the Pfam database yielded 11,533 unique Pfam domains^31^ (“Methods”). To find domains relevant to PCN, we first performed a point-biserial correlation analysis across all domains, followed by multiple testing correction (Benjamini–Hochberg false discovery rate (FDR), *q* < 0.05). This analysis revealed 1288 (~11.2%) domains significantly associated with PCN, indicating their potential roles in copy number regulation (Fig. 2A). We created binary features for each plasmid based on the presence or absence of these domains. Besides domains, we also incorporated other features in our model, including plasmid length, host chromosomal length, and plasmid *k*-mer frequencies (up to 3-mers), to construct a multi-modal feature matrix that captures both sequence composition and functional properties.

To assess the impact of feature selection on predictive accuracy, we systematically tested various feature combinations. For each combination, the model was trained and evaluated through 10 independent training-test splits at a 4:1 ratio (“Methods”). The results showed that models integrating multiple feature types consistently outperformed single-feature models, with the highest accuracy achieved when combining domains, plasmid length, and chromosomal length (Fig. 2B, Supplementary Fig. S2). Consistent with our mechanistic modeling of the power-law scaling, plasmid length alone had only moderate predictive power, reinforcing the necessity of a multi-feature approach for reliable PCN estimation (Fig. 2B, Supplementary Fig. S2).

Given the different availability of plasmid host information in various applications, we chose two models for different use cases. The first model (*R*² ~ 0.72) incorporates plasmid domains, plasmid *k*-mers, plasmid length and chromosomal length, making it suitable for studies with accessible host genome data (Fig. 2C). However, in scenarios like metagenomics, host chromosomal information is often unavailable. To address this, we chose a simplified, chromosome-independent model using only plasmid-derived features (plasmid domains, plasmid *k*-mers, and plasmid length), which maintained high accuracy (*R*² ~ 0.71). This flexibility ensures broad applicability, enabling PCN prediction even in datasets where plasmid hosts are unknown. Together, these models provide a powerful framework for PCN prediction in diverse contexts.

### Associations between PCN and plasmid-encoded protein domains

**Fig. 3:**
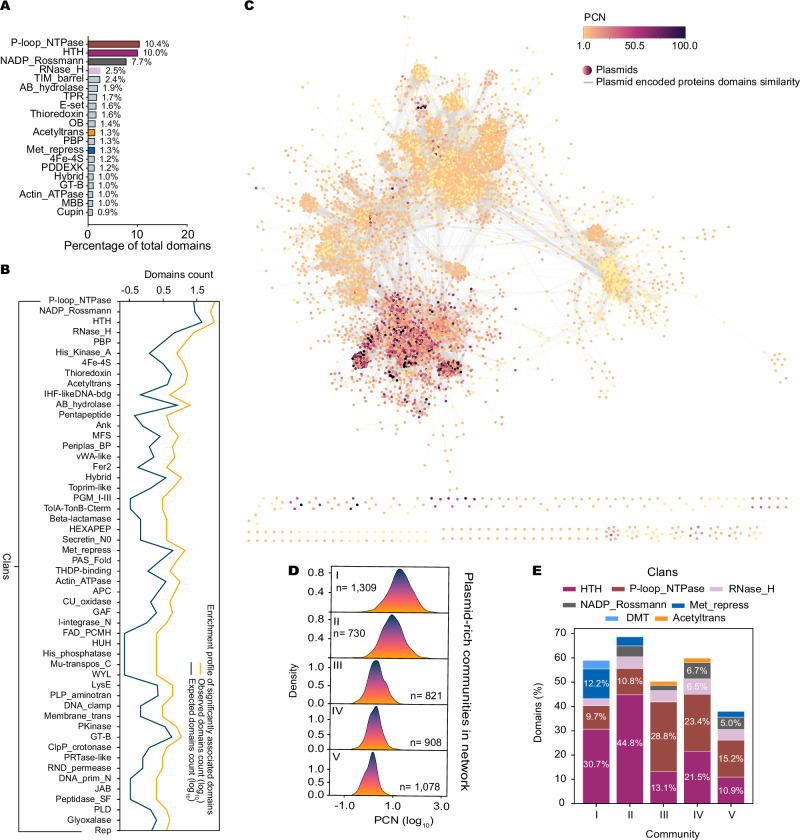
Plasmid copy number (PCN) was shaped by plasmid-encoded protein domains. **A** Fractions of the top 20 clans in the 1288 protein domains that are significantly associated with PCNs. **B** Enrichments of different clans relative to their baseline abundances across all plasmid-encoded domains. The expected domain count in each clan was calculated under the null hypothesis of random distribution using the following parameters: the total number of selected domains (1288), the total number of domains in the dataset (11,533), and the total number of domains in each clan. Statistical significance of observed vs expected distributions was calculated by one-sided hypergeometric tests (*p* < 0.05 threshold). Only clans showing significant enrichment are shown here. Source data are provided as a Source Data file. **C** Domain similarity network of 10,707 plasmids, colored by PCN (darker indicates higher PCN), with edges representing cosine similarity (cutoff = 0.5) in domain composition. **D** PCN distribution across different plasmid communities. Kernel density estimation plots of PCNs (logarithmic scale) in the five major plasmid clusters are shown (*n* = community size). **E** Functional specialization of plasmid communities. Stacked bar plot showing clan composition across communities. Source data are provided as a Source Data file.

Evolutionary related protein domains can be organized into broader groups known as clans^33^. Domains within a clan typically share sequence, structural or functional similarities. Among the domains significantly associated with PCN, the most prevalent clans are *P*-loop NTPase, helix-turn-helix (HTH), and NADP_Rossmann, together accounting for 28% of all identified domains (Fig. 3A). Notably, these clans are significantly enriched relative to their baseline abundances across all plasmid-encoded domains (Fig. 3B). The P-loop NTPase clan, mainly composed of Type III restriction subunits (PF04851) and FtsK/SpoIIIE family proteins (PF01580), drives ATP-dependent DNA processing (GO:0005524)^34^. It performs critical replication and segregation functions through helicase activity and strand translocation (GO:0003677). Meanwhile, HTH domains, especially Sigma-70 factors (PF08281) and HTH AraC-family regulators (PF00165), regulate gene expression adaptively via sequence-specific DNA binding (GO:0043565) and transcription initiation (GO:0006352), allowing plasmids to dynamically respond to antibiotic pressure or host environmental shifts^35–37^. The NADP_Rossmann clan, rich in oxidoreductases like 3-beta hydroxysteroid dehydrogenase (3Beta_HSD) (PF01073), sustains plasmid viability under stress by mediating NADP-dependent redox reactions (GO:0016616) and metabolic integration with host pathways^38^. Together, these clans form an optimized functional triad: P-loop NTPases ensure faithful inheritance, HTH domains adjust gene expression for rapid adaptation, and NADP_Rossmann enzymes maintain metabolic compatibility in various niches. This architecture underscores how plasmids balance replication fidelity, transcriptional flexibility and physiological resilience to thrive as vectors of horizontal gene transfer.

Plasmid-encoded domains allow us to construct a network that visualizes the functional similarities among plasmids. For every plasmid pair, we calculated the cosine similarity of their encoded domains (“Methods”). After applying a stringent similarity threshold (cosine similarity ≥ 0.5), we created a network with 10,707 nodes (each representing a plasmid) and 1,039,492 edges connecting functionally related plasmids. This network allows us to examine the distribution patterns of plasmid functions and the relationships between plasmid functional content and copy number (Fig. 3C, Supplementary Fig. S3A).

In the network, plasmids formed tightly interconnected clusters, and this pattern persisted even under more stringent similarity thresholds (cosine similarity ≥ 0.7; Supplementary Fig. S3B). Notably, low-copy (<5 copies per cell) and high-copy plasmids (>20 copies per cell) were located in different regions of the network with little overlap. Applying the Louvain community detection algorithm^39^, 112 functionally distinct clusters were identified within the network (Supplementary Fig. S3C). Among them, the five largest clusters (I–V), collectively comprising 4846 plasmids (45% of the total network), exhibited clear differences in both PCN distributions and domain compositions (Fig. 3D, E). Clusters I and II, the high-PCN clusters, were highly enriched in HTH domains (30.7% and 44.8%, respectively), while clusters III, IV and V were dominated by low-copy plasmids with greatest enrichment of P-loop NTPase clans (28.8%, 23.4% and 15.2% respectively) (Fig. 3E). Together, these analyses create a comprehensive map of PCN regulation where copy number variation results from functional constraints on conserved protein domains.

### Model-based prediction identifies high-copy, ARG-rich plasmids in clinical pathogens

Plasmids are key vehicles of horizontal gene transfer, mediating the spread of ARGs and virulence factors among microbes^1^. Plasmids in prokaryotic pathogens substantially contribute to their drug resistance^40^. Predicting the copy numbers of these clinical plasmids is crucial for understanding the dosage and transfer potential of pathogens’ ARGs and is significant for the surveillance and risk assessment of ARG outbreaks. However, due to the lack of a predictive framework, the copy number diversity of pathogenic plasmids remains largely unknown.

To address this, we applied our machine-learning approach to the plasmids from NCBI’s Pathogen Detection database, a comprehensive resource of microbial pathogen genomes from food, environment and patient samples^41^. We retrieved the genomes of all fully sequenced prokaryotic pathogens available as of May 2, 2025. These genomes cover 88 pathogen species, with *S. enterica*, *E. coli* and *Shigella* being the most represented. From the 15,855 genome assemblies obtained, we identified 30,254 plasmids, with ~67.3% of the genomes carrying at least one plasmid.

**Fig. 4:**
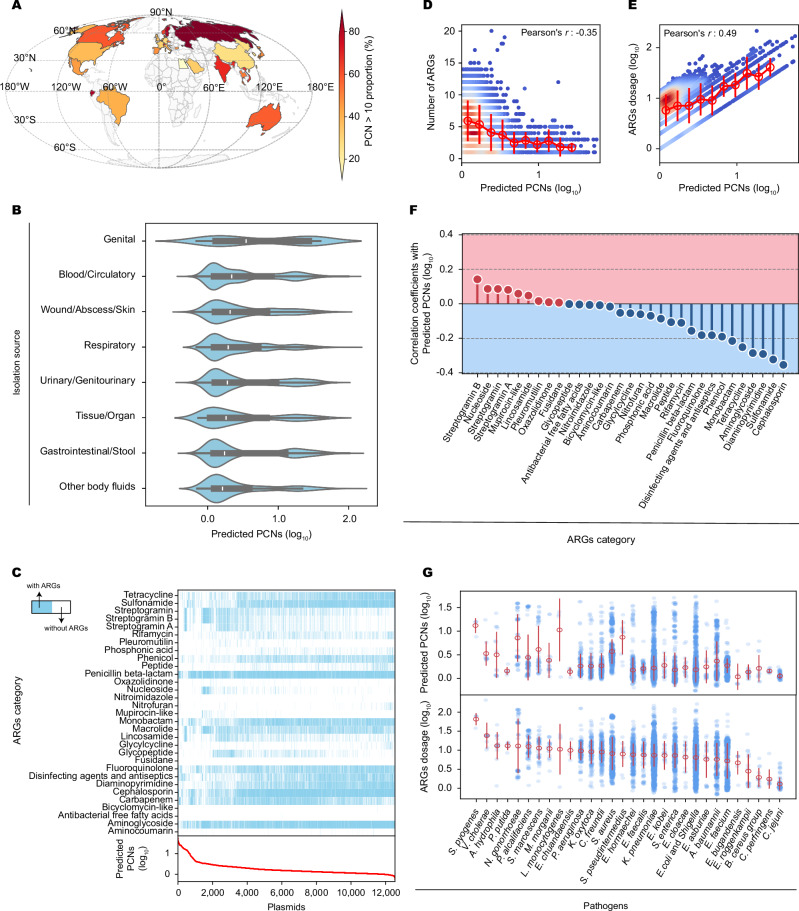
Distribution patterns of predicted plasmid copy number (PCNs) and antibiotic resistance gene (ARG) dosage in clinical plasmids. **A** Geological map showing the proportion of plasmids with PCN > 10 in different countries. **B** Distribution of predicted PCNs across various human isolation sources. The box plot inside each violin represents the median and interquartile range (IQR) of predicted PCNs and whiskers extending to 1.5 × IQR. The median line for each group is highlighted within the box plot. Groups are sorted by the median predicted PCN in descending order (*n* = 18,808 plasmids across 8 isolation source groups). **C** Distribution of different ARGs in clinical plasmids. The rows represent different ARG categories, while the columns represent individual plasmids. The red curve at the bottom indicates the predicted PCNs across plasmids. **D** Correlation between predicted PCNs and the number of ARGs per plasmid copy (*n* = 12,546 plasmids). Color shading represents point density. The PCN range was divided into equal-width bins. Bar plots represent the mean ± S.D. of ARG numbers per plasmid within each bin. **E** Correlation between predicted PCNs and ARG dosage (*n* = 12,546 plasmids). Color shading represents point density. The bar plots represent the binned average ± standard deviations. **F** The point-biserial correlation coefficient between predicted PCN and the presence/absence of the ARGs per plasmid. Red and blue lines represent positive and negative correlations, respectively. ARG categories are sorted by correlation coefficient. Source data are provided as a Source Data file. **G** Distribution of predicted PCNs and plasmid ARG dosages across various pathogens, with error bars representing the standard deviation around the mean within each pathogen group (*n* = 12,513 plasmids across 30 groups).

We applied the full-context model to predict the copy numbers of all pathogenic plasmids by integrating plasmid-encoded domains, *k*-mers, plasmid length and chromosomal length. The predicted PCNs, reaching up to 128, showed substantial diversity and maintained a power-law relationship with plasmid size, characterized by a scaling coefficient of −0.73 (Supplementary Fig. S4A). The close agreement with the coefficient derived from the training dataset (Fig. 1A) underscores the robustness of our machine learning model. Moreover, we observed a geographic pattern, where high-copy-number plasmids (PCN > 10) were overrepresented in samples from Russia, Sweden and India (Fig. 4A, Supplementary Fig. S4B). Further stratification by anatomical isolation sites showed that pathogenic plasmids from human genital samples had elevated PCN (Fig. 4B). This trend is likely due to species composition differences across niches rather than niche-specific variations within species. Analysis of taxonomic composition across clinical niches, stratified by body site and geographic region, reveals clear taxonomic trends (Supplementary Fig. S4C, D). For instance, *Neisseria gonorrhoeae* was enriched in genital samples, which plausibly contributes to the elevated PCN observed in this niche. *N. gonorrhoeae* is also enriched in samples from Russia relative to other regions, helping to explain the high PCN observed there (Supplementary Fig. S4C–G).

To examine the association between PCN and ARG carriage, we annotated ARGs in all pathogenic plasmids, identifying 12,546 plasmids harboring ARGs from 31 drug classes^42^ (Fig. 4C, Supplementary Fig. S5A; “Methods”). The prevalence of different ARG categories varied across isolation sources, with penicillin beta-lactam, cephalosporin, and aminoglycoside resistance genes being most common in most clinical sample types (Supplementary Fig. S5B). We then calculated ARG richness and dosage for each plasmid, with dosage defined as richness multiplied by PCN. High-copy plasmids showed lower ARG richness (Pearson’s *r* = −0.35, Fig. 4D), consistent with their typically smaller sizes. Conversely, ARG dosage correlated positively with PCN (Pearson’s *r* = 0.49) (Fig. 4E), suggesting that while high-copy plasmids carry fewer distinct resistance genes per plasmid, their elevated copy number compensates for size constraints, amplifying overall ARG abundance. Analyses across body sites confirmed these trends (Supplementary Fig. S5C, D).

While overall ARG richness exhibited a negative correlation with PCN, ARGs from different drug classes showed various correlations. Most ARG types, especially those related to cephalosporin, sulfonamide, diaminopyrimidine and aminoglycoside, displayed negative correlations (Fig. 4F). In contrast, streptogramin-type ARGs showed weak positive correlations, indicating that these genes tend to be enriched in small high-copy-number plasmids.

Among different pathogens, *Streptococcus pyogenes*, which causes impetigo, pharyngitis, cellulitis and acute Rheumatic fever, stands out with the highest mean plasmid ARG dosage^43^ (Fig. 4G). *Vibrio cholerae*, the causative agent of cholera, and *Aeromonas hydrophila*, which causes gastroenteritis and wound infections, also rank highly^44,45^. These pathogens may pose greater risks in the evolution and dissemination of ARGs. In contrast, *Enterococcus* and *Campylobacter* plasmids display low PCN and ARG dosage, potentially due to chromosomal integration of resistance genes or ecological niches with reduced selection for plasmid-mediated resistance. These findings offer critical insights for risk evaluation and ARG surveillance in prokaryotic pathogens.

### PCN landscape across ecosystems

To investigate PCN distribution across different microbial ecosystems, we further applied our machine learning framework to the IMG/PR dataset, which contains the most extensive collection of plasmid sequences and associated metadata from diverse environments^14^. As many plasmids in the database were derived from metagenomes without validated host organisms, we used the chromosome-independent model relying solely on plasmid-derived features (domains, plasmid *k*-mers, and plasmid length) (Supplementary Fig. S6A). For robust analysis, we restricted our predictions to the 136,195 fully sequenced plasmids labeled as ‘putatively complete’.

**Fig. 5:**
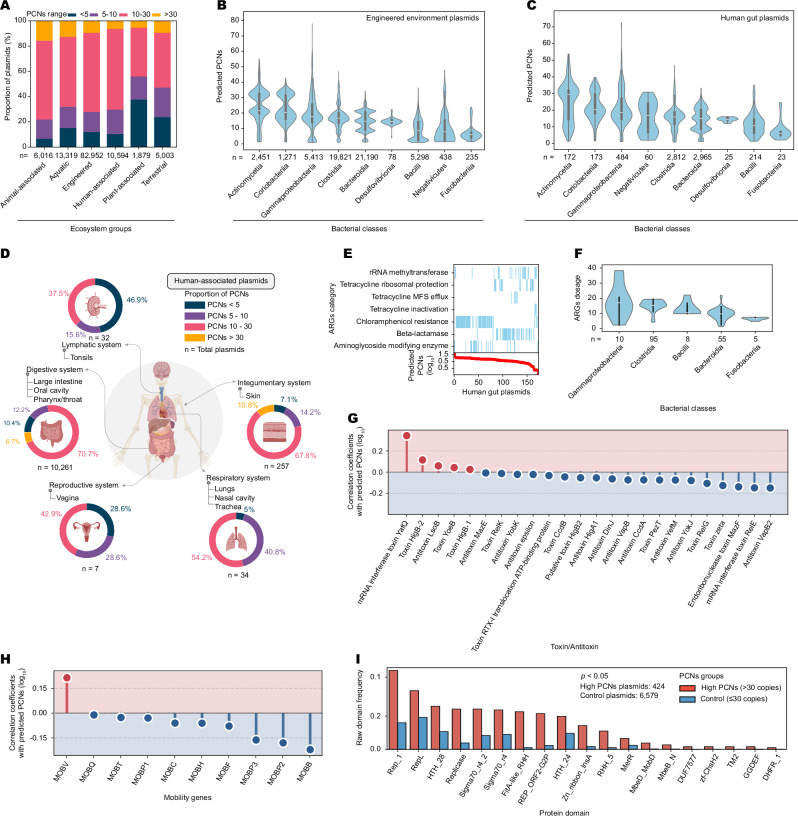
Predicted plasmid copy number (PCN) distribution patterns across different ecosystems. **A** Proportion of plasmids within different PCN ranges in various ecosystem groups. Here *n* stands for sample size. **B** Taxonomic distribution of predicted PCNs in the engineered environment, with *n* standing for sample size. The box plot inside each violin represents the median (center line), interquartile range (IQR; box bounds from 25th to 75th percentile), and whiskers extending to 1.5 × IQR. **C** Taxonomic distribution of predicted PCNs in the human gut microbiome. The box plot inside each violin represents the median (center line), interquartile range (IQR; box bounds from 25th to 75th percentile), and whiskers extending to 1.5 × IQR. **D** Pie charts showing the proportion of plasmids within different PCN ranges in different human body systems. The figure was created in BioRender. Wang, T. (2026) https://BioRender.com/wcaten9. Source data are provided as a Source Data file. **E** Distribution of antibiotic resistance genes (ARGs) across plasmids (sorted by predicted PCN) in the human gut microbiome. Rows represent different ARG categories, and columns represent individual plasmids. The red curve at the bottom shows the overall distribution of predicted PCNs across plasmids. **F** Distribution of ARG dosage in human gut plasmids across bacterial classes. Boxplots indicate the median (central line), interquartile range and whiskers extending to 1.5 × IQR with *n* representing the sample size. **G**, **H** Stem plots illustrate the correlation between toxin/antitoxin genes (**G**) and mobility genes (**H**) with predicted PCN. Point-Biserial correlation was used for correlation calculation. Source data are provided as a Source Data file. **I** Frequency of protein domains in high-copy plasmids (PCN > 30, shown in red) compared to other plasmids (PCN ≤ 30, shown in blue). Only domains significantly enriched in high-PCN plasmids are shown (one-sided Mann-Whitney *U*-test, false discovery rate (FDR)-corrected *p* < 0.05, fold enrichment > 1). Source data are provided in the Source Data file.

Predicted PCNs followed a power-law relationship with plasmid size, with a scaling coefficient of –0.90, closely matching that from the training dataset (Supplementary Fig. S6B). Our analysis revealed that while engineered ecosystems (wastewater treatment plants, bioreactors) contributed the largest fraction of plasmids (60% of the total), animal-associated environments showed the highest proportion ( ≈ 80%) of high-copy-number plasmids (PCN > 10). In contrast, plasmids from plant-associated and terrestrial niches were predominantly low-copy (PCN < 10), with mean copy numbers of ~10 and 13, respectively (Fig. 5A). Taxonomic stratification showed PCN variations across prokaryotic classes. Among plasmids from engineered environments, including bioreactors, wastewater treatment plants, and artificial ecosystems (Fig. 5B), *Actinomycetia* had the highest median PCN (≈20 copies), followed by *Coriobacteria* (≈ 18 copies) and *Gammaproteobacteria* (≈ 15 copies). At the low end, *Bacilli* (≈ 10 copies) and *Fusobacteriia* (≈ 8 copies) had the lowest median copy numbers. This pattern also held for plasmids in the human gut microbiome (Fig. 5C).

In human-associated environments, PCNs varied significantly across anatomical sites. The digestive system (the largest plasmid source in humans) and integumentary systems emerged as the epicenter of high-copy plasmids, with 77.4% and 78.6% exceeding PCN > 10, respectively (Fig. 5D). Conversely, the lymphatic system was predominantly low-copy, with 62.5% of plasmids below PCN 10. These patterns likely reflect the niche-specific effects across body sites.

Human gut microbiome is a major reservoir of ARGs^46^. Multiple types of ARGs were detected in the human gut plasmidome (Fig. 5E). Chloramphenicol resistance loci are primarily found on mid-to-high copy plasmids. By contrast, β-lactamases are mainly present on low-copy-number plasmids. When stratified by bacterial class, *Gammaproteobacteria* carry the highest ARG dosage, followed by *Clostridia* and *Bacilli* (Fig. 5F).

Further analysis identified the associations between PCN and other plasmid traits such as toxicity and mobility (Fig. 5G, H). Toxins are the primary pathogenicity factors produced by many bacteria^47^. Many toxins are paired with antitoxins that counteract their toxic effects. Our analysis suggested that in the human gut microbiome, many toxin-antitoxin-related genes, especially mRNA interferase toxin *yafQ*, were associated with PCN^48^ (Fig. 5G, Supplementary Fig. S6C). Analysis of conjugation systems also revealed complex relationships with PCN. While most mobility genes showed neutral or negative associations with predicted PCN, the MOBV family demonstrated a positive correlation (Pearson’s *r* = 0.20) (Fig. 5H, Supplementary Fig. S6D). These findings indicate that different conjugation systems have evolved for plasmids with different copy numbers.

Many plasmids in human gut microbiome are predicted to have very high copy numbers (PCN > 30). To understand the potential mechanisms underlying their high copy number, we compared the protein domains encoded by these plasmids with those encoded by other plasmids. Our analysis revealed two key functional shifts. The replication machinery showed notable enrichment, featuring Replicase domains (6.2-fold increase) along with Rep_1 (2.9-fold; GO:0003677 DNA binding, GO:0006260 DNA replication) and RepL (1.8-fold; GO:0006260 DNA replication, GO:0006276 plasmid maintenance). Transcriptional regulation domains were also prominent, with Sigma70_r4_2 (2.89-fold; GO:0003677 DNA binding, GO:0003700 transcription factor activity, GO:0016987 sigma factor activity) and Sigma70_r4 (2.63-fold; GO:0003700 transcription factor activity, GO:0006352 transcription initiation, GO:0006355 transcriptional regulation)^35^ showing strong enrichment (Fig. 5I). These correlative patterns suggest a candidate hypothesis that high-copy plasmids might have evolved gut-specific strategies to tightly regulate replication initiation in response to fluctuating nutrient availability and rapidly modulate gene expression to adapt to host dietary changes or immune pressures. If validated experimentally, such mechanisms could not only enhance plasmid persistence in the gut ecosystem but may also facilitate the spread of adaptive traits, including antibiotic resistance genes, among gut microbiota.

## Discussion

Our study integrates theory and machine learning to address key conceptual and practical gaps in understanding plasmid copy number control. Recent foundational studies established empirical patterns, most notably the inverse power-law relationship between plasmid size and PCN, but the mechanistic and evolutionary origin of such patterns remains unclear^2,3^. Our theoretical model addresses this gap by showing that the size-PCN tradeoff can emerge naturally from multi-level selection: host-level pressure to minimize metabolic burden and plasmid-level selection to maintain replication competitiveness. While intentionally simple, this framework captures the core dynamics and predicts additional relationships (e.g., between chromosome size and plasmid DNA content) that lack prior mechanistic interpretation.

However, this model necessarily overlooks many complexities, such as the functional diversity of plasmid-encoded proteins. Incorporating such factors into a cohesive mechanistic framework is challenging, as the combinatorial parameter space quickly becomes intractable. To address this limitation, we developed a data-driven approach that explicitly incorporates heterogeneous plasmid features. This framework significantly improved prediction accuracy and directly links molecular features to copy number variation, with plasmid-encoded domains emerging as key PCN predictors. Our machine learning framework complements mechanistic modeling by bridging biological complexity with computational efficiency, thus enabling rapid and large-scale copy number predictions of plasmids sampled from microbiomes across the world. Our work thus provides both mechanistic foundations for universal scaling laws and extends their application to ecosystem-scale prediction and biological interpretation. In addition, our framework provides a null baseline for the expected plasmid copy numbers. Measured plasmid copy numbers that significantly exceed this null baseline would evidence strong selection for exceptional plasmid copy numbers in particular contexts, such as very recent exposure to strong antibiotic dosages or other stresses^9,49^.

The performance of our machine learning framework is inherently constrained by the noise in the training dataset^3^. For instance, PCN estimations rely on coverage-based methods, which intrinsically introduce more errors for small plasmids. Additionally, the dataset is skewed, with *Enterobacteriaceae* (43%) and human-associated environments (37.7%) overrepresented, while archaea (0.23%) and engineered ecosystems (0.2%) are underrepresented, limiting extrapolation. We systematically evaluated these limitations through cross-domain testing. Models trained on bacteria show reduced performance on archaea (*R²* = 0.451, Supplementary Fig. S8A), while models trained on human-associated plasmids exhibit environment-dependent accuracy (Supplementary Fig. S8B, C). These cross-domain performance differences reflect genuine biological variation. While the inverse size-PCN relationship persists, scaling exponents vary across different environments (Supplementary Fig. S8D). Future improvements would benefit from expanding validated PCN datasets for underrepresented taxa and environments.

Environmental factors can influence PCN, too. For instance, growth conditions (such as temperature, pH and nutrients) can alter the copy number of plasmids^50,51^. PCN can also vary as bacteria transition from logarithmic to the stationary growth phase^52^. Additionally, PCN heterogeneity can arise even in clonal populations^11^.

The same plasmid may exhibit different copy numbers in different hosts, further complicating PCN prediction. For instance, the PCN of the pCff119 plasmid varied significantly among 25 isolates of the bacterium *Curtobacterium flaccumfaciens* pv. *flaccumfaciens*^53^. Another example is the plasmid pB1000 (usually found in *Pasteurellaceae*), which adapts to the *E. coli* host with increased copy number^54^. Host physiology, cell cycle regulation, and metabolic capacity can, in principle, influence PCN, and these interactions are a vital part of plasmid ecology. This work did not intend to dismiss these effects; rather, our work aimed to address a common practical challenge: in most datasets, detailed information about the host’s physiological state is unavailable. Our methodological focus, therefore, was to assess the predictive power of plasmid-encoded features in the absence of host data—a scenario that reflects the reality of metagenomic studies. Our analyses were designed to quantify how much of the observable PCN variation can be attributed to the plasmid itself. The empirical results from our study, corroborated by recent large-scale work^2^, suggest that host-dependent variation, while biologically meaningful in specific instances, is not the primary source of PCN variance across the plasmid landscape. For instance, the inclusion of the host genus in our model yielded a very modest improvement in predictive power (ΔR² ≈ 0.006, Supplementary Fig. S2D). Furthermore, our systematic analysis across 6286 plasmids found that most replicon types (88.7%) exhibited no statistically significant PCN variation across different host genera (Supplementary Fig. S7A). We note that host taxonomy at the genus level is an imperfect proxy for host physiology, and the limited contribution of host features in our model likely reflects this coarse resolution rather than a true absence of host effects. Plasmid copy number should be interpreted as an emergent property of host–plasmid–environment interactions, with finer-scale physiological and environmental variation remaining unresolved in current large-scale datasets.

Nevertheless, these findings offer a valuable ecological perspective. By establishing a robust baseline prediction for PCN, our model provides an ecologically meaningful null expectation. Significant deviations from this baseline in specific host-plasmid combinations then become interpretable as signals of unique adaptation, stress, or interaction. In the absence of high-resolution host physiological data, some PCN variation within the same plasmid family may also reflect unresolved host effects. Our approach allows researchers to move from a decontextualized single PCN value to a framework where observed PCN can be evaluated relative to its plasmid-determined expectation, thereby contextualizing it within a potential host environment.

In this work, we relied on sequence features for prediction, while disregarding the confounding factors listed above due to their absence in published sequencing data and a lack of standardized growth conditions across thousands of samples worldwide. This limitation may contribute to the discrepancies between predicted and actual PCNs. Nevertheless, the relatively high accuracy of our machine learning predictions indicates that our approach identifies the most critical features governing PCN control, highlighting the fundamental role of plasmid sequence in determining copy number.

Replicon family classification captures broad replication strategies, providing categorical labels (e.g., low- and high-copy families) to different plasmids. However, many ecological and functional outcomes—such as gene dosage and antibiotic resistance amplification—depend on continuous variation in plasmid copy number that cannot be resolved by categorical labels alone. Quantitative PCN prediction, therefore, provides an essential layer of resolution, enabling more precise linkage between plasmid biology and ecological or clinical phenotypes.

While plasmids are well-known as the main carriers of ARGs, the relative contribution of small, high-copy plasmids versus large, low-copy plasmids to ARG dissemination remains unclear. Our method enables the estimation of ARG dosage per plasmid, facilitating risk assessment for plasmid-mediated ARG dissemination. Our results indicate that although high-copy plasmids harbor fewer ARGs due to the size-copy number tradeoff, their elevated copy number may compensate for size limitations, potentially leading to higher overall ARG abundance. This suggests that small, high-copy plasmids may amplify overall ARG dosage, while large low-copy plasmids act as key hubs for ARG dissemination^49,55^. These findings provide key insights for the surveillance, control, and reversal of plasmid-mediated ARG transfer.

Application of our framework to metagenomic plasmids revealed environment-specific PCN patterns. The systematic differences in PCN distributions, plasmid domain composition and ARG gene dosage across environments reflect real, niche-specific signatures in the plasmid pool. These signatures likely arise from the interplay of plasmid-intrinsic replication strategies and broad environmental selection pressures. Importantly, identified PCN “hotspots” should not be construed as evidence of environment-specific replication behavior independent of host taxonomy. Instead, they represent environments where plasmids with genetic architectures linked to higher copy numbers are preferentially enriched. Current limitations in host taxonomic resolution necessarily constrain definitive ecological interpretation. Therefore, these findings are primarily hypothesis-generating, establishing a quantitative, plasmid-centric foundation for future studies that incorporate host-resolved taxonomic and physiological data.

## Methods

This study involved computational analysis of publicly available genomic data and did not involve human subjects, animal experimentation, or clinical interventions. Therefore, no ethics approval was required.

### Data collection and processing

We analyzed a comprehensive dataset comprising 11,338 fully assembled plasmid sequences from 4,317 prokaryotic genomes with copy number estimates from Maddamsetti *et al*^3^. The PCNs were quantified using the Probabilistic Iterative Read Assignment (PIRA) method, which combines pseudoalignment with iterative refinement for accurate PCN estimation across large sequencing datasets. Following their pipeline, we retrieved complete plasmid assemblies and associated metadata using NCBI Datasets command-line tools, retaining only those entries labeled as “plasmid”. Most plasmids are from natural environments, while some are explicitly labeled as ‘engineered’. These plasmids were constructed or modified in laboratories. Taxonomic information of plasmid hosts is also provided in the associated metadata.

### Feature extraction

For each plasmid in the dataset, we first calculated the plasmid length and, when available, the chromosomal length of the host genome. To characterize sequence composition, we also calculated plasmids' *k*-mer frequencies for *k* = 1, 2 and 3. In order to derive the functional features, protein-coding sequences of the plasmids were identified using Prodigal (v2.6.3)^32^. Domain annotation of these protein coding sequences was performed against the Pfam database (v37.1) using HMMER’s HMMScan (v3.4)^56^, applying stringent *E*-value thresholds (sequence-level: ≤ 0.001; domain-level: ≤ 0.001), to ensure high-confidence domain annotations. This process identified 11,533 unique Pfam domains. Plasmids without detectable domains were excluded from further analysis (retaining 11,051 plasmids), and each domain was represented as a binary feature indicating its presence (1) or absence (0) for the downstream analysis.

To identify domains significantly associated with PCN, we calculated point-biserial correlation coefficients between each binary domain feature and PCN. We adjusted for multiple testing using the Benjamini-Hochberg false discovery rate correction, retaining only domains with *q*-values < 0.05. This statistical filtering reduced the genomic feature set to 1288 domains (approximately 11.2% of the total). Learning curve analysis confirmed that including additional domains beyond this threshold did not improve model performance (Supplementary Fig. S9B), demonstrating that significance-based filtering captures almost all predictive protein domains. We subsequently characterized the domains using Pfam-to-GO mappings^35^ (version: 2025/04/29 15:50:00) to investigate their potential biological roles in plasmid maintenance and replication.

### Development of machine learning framework

To establish an optimal framework for PCN prediction, we first evaluated the relative contributions of different feature combinations through a tiered approach (Fig. 2A, B). We trained parallel models across all possible combinations of four feature categories: (1) the 1288 statistically significant Pfam domains, (2) absolute *k*-mer frequencies, (3) plasmid length, and (4) host chromosomal length, where available.

The log(1 + PCN) transformation of PCNs was applied to address the intrinsic statistical properties of plasmid copy number data. PCN values in the dataset span several orders of magnitude, from a few to several hundred, resulting in a strongly right-skewed distribution. The transformation compresses the dynamic range, making the variance more homogeneous across the scale of PCN values. In addition, the transformation linearizes relationships that are inherently multiplicative, such as the power-law relationship. The addition of “+1” is a standard continuity correction that prevents taking the logarithm of zero, which would be mathematically undefined, while having a negligible impact on the transformed values for all PCN > 1. This approach aligns with best practices for modeling count-based genomic data and has been shown to effectively preserve biological signal while improving model fit^57^. All predicted values were subsequently back-transformed to calculate PCNs.

Each feature combination was evaluated across 10 independent train-test splits (80/20 ratio), with each replicate initialized using a unique random seed. This design mitigates the influence of stochastic training variability and data-partitioning effects. Performance metrics (R², mean square error (MSE), and Spearman’s *ρ*) are reported as mean ± S.D. across the 10 replicates, enabling direct comparison between simplified single-feature models and comprehensive multi-feature integrations.

We pre-evaluated various regression algorithms using default parameters, including scikit-learn v1.5.2 simple linear regression^58^, random forest regressor^58^, and XGBoost (v2.1.1)^59^. Consistent preliminary results demonstrated random forest’s superior performance across all feature combinations (Supplementary Fig. S2C), leading to its selection as our base algorithm. Subsequent hyperparameter tuning employed a two-phase strategy: an initial broad random search cross-validation across wide parameter ranges (documented in code repository), followed by focused grid search cross-validation around optimal candidates identified during the exploratory phase. The final optimal hyperparameters were: n_estimators = 113, min_samples_split = 15, min_samples_leaf = 1, max_features = 0.4, max_depth = 290.

In our feature-ablation analysis, the highest predictive accuracy was achieved by a model using only protein domains, plasmid length, and chromosomal length. For downstream application, however, our final models were designed to prioritize generalizability and biological completeness, leading us to include short *k*-mer (*k* = 1–3) features. K-mers provide complementary, annotation-agnostic information that captures sequence-level regulatory determinants—particularly in non-coding regions—which are not encoded by protein-domain annotations and can vary even among plasmids with identical replication machinery. Including *k*-mers ensures robust performance on novel or incompletely annotated plasmids from metagenomes, a core application scenario. Here, model selection was guided by the principle of maximizing practical utility and biological insight for ecological inference, alongside predictive accuracy. Therefore, we accepted an almost negligible reduction in peak theoretical performance on a clean benchmark to gain robustness for applied use.

To ensure a comprehensive assessment of potentially informative predictors, we extended the feature space to include higher-order *k*-mer frequencies (*k* = 4–6), GC%, and genus information. To control feature dimensionality in taxonomic variables, genera with ≥1.0% dataset representation (*n* = 13) were retained as individual predictors, while 286 low-frequency genera were aggregated into a single “Other” category. The inclusion of genus information led to a statistically significant but modest improvement in performance (ΔR² ≈ 0.006, Supplementary Fig. S2D), indicating that while host identity contributes additional information, the majority of explainable PCN variance is already captured by plasmid-encoded molecular features. Our evaluation also revealed that *k*-mers of length 1–3 achieved optimal predictive performance (*R²* = 0.738 ± 0.013), whereas longer *k*-mers resulted in performance degradation, likely due to feature space expansion and increased sparsity (Supplementary Fig. S2D). GC content (GC%) provided gains comparable to short *k*-mers when combined with other features, but substantially underperformed when used in isolation (Supplementary Fig. S2D, E). Based on these results, we used 1, 2 and 3-mers in the final models to capture sequence-level information from non-coding regions, including replication origins and regulatory motifs not represented by domain annotations^12,60^.

Practical considerations guided the development of two specialized predictors: (1) a full context model incorporating all four feature types (domains, *k*-mers, plasmid length, chromosomal length) for scenarios with available host genomic data; (2) a plasmid-centric model utilizing only plasmid-derived features (domains, *k*-mers, plasmid length) for broader applicability in metagenomic contexts. In both models, the target variable (PCN) was log1p transformed. Both architectures demonstrated high accuracy, with complete implementation details and datasets publicly available at our GitHub repository.

To assess the robustness of our method to exceptionally large plasmids, where the plasmid-chromosome boundary may be ambiguous, we performed sensitivity analysis by retraining the model before and after excluding these plasmids ( > 500 kb; *n* = 189; 1.7% of the dataset, including the three >2 Mb elements). Model performance remained highly stable after filtering (*R²* = 0.736 ± 0.014) compared to the full dataset (*R²* = 0.746 ± 0.017; ΔR² = 0.010), and predicted PCNs were strongly correlated between the two analyses (Pearson’s *r* = 0.99) and key conclusions remained unchanged (Supplementary Fig. S2G, H), confirming that our results are not driven by edge cases at the upper size limit.

### Network analysis

To systematically investigate relationships between plasmid-encoded protein domains and copy number variation, we implemented a multi-stage computational pipeline. First, we quantified pairwise similarity between all plasmids based on their binary-encoded domain profiles, where each plasmid was represented as a vector indicating the presence (1) or absence (0) of specific protein domains. We computed cosine similarity scores of all plasmid pairs using scikit-learn’s implementation^58^, which measures the cosine of the angle between vectors to determine their similarity in domain composition while inherently normalizing for differences in plasmid size and domain richness. This produced a symmetric similarity matrix where values ranged from 0 (completely dissimilar) to 1 (identical domain composition).

For network analysis, we established a conservative similarity threshold of ≥0.5 to focus on biologically meaningful relationships, constructing an undirected weighted network where nodes represent plasmids and edges connect plasmids meeting this similarity criterion. The network was imported into Cytoscape (v3.10.3) for visualization^61^, with nodes colored by PCN values using a continuous gradient scale to enable visual assessment of PCN distribution patterns.

To identify functionally related plasmid clusters, we applied the Louvain community detection algorithm to this network^39^. This method iteratively optimizes modularity, grouping plasmids into communities that maximize intra-cluster connections while minimizing inter-cluster edges, resulting in the identification of 112 communities. Robustness of the community detection was confirmed using the Leiden algorithm^62^, which yielded nearly identical results (112 communities, modularity = 0.8055 vs 0.8054 for Louvain). Community assignments showed 99.5% average overlap (Jaccard similarity), demonstrating that the inferred community structure is not sensitive to algorithm choice (Supplementary Fig. S9C).

For downstream analysis, we focused on the five largest communities, comparing their genetic composition and PCN distributions to investigate potential relationships between domain content and copy number variation. Throughout this process, we maintained a clear separation between the quantitative network analysis (performed programmatically) and qualitative visualization (conducted in Cytoscape), ensuring methodological rigor while enabling biological interpretation of the complex network patterns.

### Plasmid replicon typing

Plasmid replicon typing was performed using MOB-typer from the MOB-suite tool (v3.1.0)^63^, which assigns replication types based on curated databases of plasmid relaxase and replication initiator proteins. MOB-typer was run with default parameters (minimum *e*-value threshold = 10^−5^, minimum sequence identity = 80% and minimum alignment coverage = 80%). This analysis assigned replicon types to 8471 of the 11,051 plasmids in our dataset; the remaining plasmids either lacked detectable replicon markers or fell below confidence thresholds.

To statistically evaluate whether PCN depends on the host background for a given replicon type, we first identified replicon types suitable for cross-host comparison. We filtered for replicon types that were present in plasmids isolated from two or more distinct bacterial genera, ensuring that each test compared PCN distributions across genuinely different hosts. This filtering yielded 150 testable replicon types. For each of these replicon types, we compared PCN distributions across the different host genera using the non-parametric Kruskal–Wallis *H*-test, which is robust to non-normal distributions and unequal sample sizes. To control the false discovery rate across the 150 simultaneous tests, we applied Benjamini–Hochberg correction with a false discovery rate (FDR) of 5%. Replicon types with an adjusted *p*-value < 0.05 were classified as host-dependent, indicating significant PCN variation across hosts; those with an adjusted *p*-value ≥ 0.05 were classified as host-independent.

To quantify the degree of PCN variation within each replicon family (e.g., Inc groups, Col groups), we calculated the Coefficient of Quartile Variation (CQV). We restricted this analysis to replicon families present in at least two bacterial genera to ensure reliable quartile estimates. Families with CQV ≥ 0.5 were classified as high-variation, indicating substantial heterogeneity in PCN even within the same replication type.

To assess associations between protein domains and replicon families, we performed one-sided Fisher’s exact tests evaluating the over-representation of each protein domain within each family compared to all other families. The analysis included 1288 protein domains and the 64 most prevalent replicon types, which collectively cover 80% of plasmid–replicon pairs in our dataset. To ensure statistical reliability, only replicon families containing at least two plasmids were included. *P*-values were adjusted for multiple testing using the Benjamini–Hochberg FDR correction, with a significance threshold of p_adj < 0.05.

While plasmid replicon type is an important determinant of PCN^2^, our analysis demonstrates that molecular features provide significant additional predictive power (Supplementary Fig. S2F). To evaluate the contribution of our framework beyond broad family classification, we trained another model that predicts PCN based solely on replicon type. When directly compared, our integrated domain-centric model significantly outperformed this replicon-only baseline. This performance gain confirms that the detailed molecular architecture captured by our model—including protein domains, sequence composition, and size—captures the fine-scale regulatory variation that determines precise PCN, moving beyond the categorical distinctions provided by replicon family alone.

### Applications to clinical plasmids

To assess the copy number distribution of clinically relevant plasmids, we obtained genomic data from NCBI’s Pathogen Detection resource on May 02, 2025. We applied stringent filters to select only “complete” genomes from “clinical” isolates, yielding 15,855 genomic accessions. Using NCBI command-line tools, we downloaded these genomes and extracted plasmid sequences by filtering for records explicitly annotated as ‘plasmid’. This process identified 30,254 plasmids from 10,664 genome assemblies, with the remaining genomes containing no detectable plasmids.

To extract the features required for our machine learning framework, we processed these clinical plasmids through an identical bioinformatics pipeline. Protein-coding sequences were predicted using Prodigal, followed by domain annotation with HMMScan against the Pfam database, using the same versions as of model training. Of the initial 30,254 plasmids, 30,246 contained coding sequences, and we successfully identified at least one domain in 29,490 plasmids. The convergence between clinical plasmids (8960 unique domains) and our training feature set, with 1281 of 1288 statistically significant domains (99.5%) reappearing in clinical isolates, demonstrates that our feature selection captures fundamental, evolutionarily conserved plasmid elements. For PCN prediction, we constructed a feature matrix comprising plasmid lengths, host chromosomal lengths, absolute *k*-mer frequencies (*k* = 1, 2 and 3) and 1288 domains. The full context model generated predictions for log1p-transformed plasmid copy numbers (PCN), which we subsequently back-transformed to PCN values.

To investigate the links between copy number and ARG carriage, we performed comprehensive ARG annotation using the Comprehensive Antibiotic Resistance Database (CARD v4.0.0) and Resistance Gene Identifier (RGI v6.0.3)^42^. This analysis identified ARGs in 12,546 plasmids spanning 31 drug classes, enabling subsequent dose-effect analyses between predicted PCN and resistance gene carriage.

To analyze PCN distribution across isolation sources, we categorized the 18,808 plasmids with clear isolation metadata into broad anatomical groups based on source keywords. The largest groups included: Blood/Circulatory (5380 plasmids), Gastrointestinal/Stool (4795), and Urinary/Genitourinary (3799). A large proportion of remaining plasmids (10,682) either lacked clear source information or originated from non-human/animal hosts. Grouping criteria were systematically defined (e.g., ‘Gastrointestinal/Stool’ included terms like ‘feces’, ‘rectal swab’, and ‘intestinal microflora’; ‘Urinary/Genitourinary’ encompassed ‘urine’, ‘ Urinary tract infection (UTI)’, and ‘urethral swab’). This categorization enabled streamlined analysis of PCN patterns across major human anatomical sites while reducing metadata complexity.

To characterize PCN distribution around the globe, we analyzed 28,074 plasmids with country-level isolation metadata (spanning 110 countries). Applying a minimum threshold of 100 plasmids per country to ensure statistical reliability, 31 countries qualified for final analysis. High-PCN prevalence was calculated per country by dividing the count of plasmids with PCN > 10 by the total plasmids isolated from that region. Geospatial mapping was implemented using Natural Earth’s administrative boundaries (1:110 m scale, v5.1.1; downloaded from https://www.naturalearthdata.com) projected in the Robinson projection via GeoPandas.

### Application to IMG/PR dataset

We implemented a comprehensive analytical pipeline to apply our model to plasmids in the IMG/PR database. Beginning with data acquisition, we programmatically retrieved all available plasmid sequences and their associated metadata through the Joint Genome Institute (JGI) Data Portal API (https://genome.jgi.doe.gov/portal/IMG_PR). The collected metadata included essential information such as plasmid topology, ecosystem, host taxonomy, plasmid length, and functional annotations including mobility genes and antibiotic resistance determinants. To ensure data quality, we established stringent filtering criteria. We excluded sequences containing more than 5% ambiguous bases and removed all plasmids shorter than 1 kb. Additionally, we only selected those plasmids labeled as “putatively complete”. This quality control process yielded a final dataset of 154,680 high-quality plasmid sequences from an initial collection of 699,978 entries, providing a robust foundation for downstream analysis.

The feature extraction process precisely followed our established training methodology while incorporating necessary adaptations for metagenomic data. We implemented a custom Python script for *k*-mer frequency calculation. For protein-coding sequence prediction, we utilized Prodigal v2.6.3 in metagenomic mode (-p meta), specifically optimized for the fragmented and heterogeneous sequences characteristic of environmental samples. The predicted coding sequences were analyzed using the same pipeline as used in the model, identifying domains in 136,195 plasmids that met quality standards. We assessed domain conservation across plasmid populations in the IMG/PR dataset. Human gut plasmids had 70.3% of reference domains, engineered plasmids had 98.8%, and environmental plasmids showed 99.8%. These high overlap rates validate our feature selection and support the model’s broad applicability. The final implementation of our plasmid-centric model for PCN prediction across these diverse ecosystems maintained consistency with our training approach, using log1p-transformed values that were subsequently back-transformed.

Plasmids were categorized into seven ecosystem groups based on metadata annotations: Terrestrial, Engineered, Plant-associated, Aquatic, Animal-associated, Human-associated, and Microbial-associated (excluded in the following analysis due to ambiguous annotations). We employed a hierarchical classification approach, prioritizing specificity, for instance, by segregating human-associated plasmids. Subcategories were established to enhance clarity; for instance, “Engineered” refers to plasmids isolated from engineered or artificial environments such as bioreactors and wastewater systems. Plasmids that did not align with predefined categories were classified as “Uncategorized”.

To identify toxin/antitoxin-related genes in the plasmids from the human gut, we performed Prokka annotation^64^, followed by searching for the term “toxin/antitoxin” in the product descriptions. Only plasmids containing toxins or antitoxins were retained for further analysis.

We compared protein domain frequencies between high-PCN plasmids (>30 copies) and control plasmids (≤ 30 copies) using one-tailed Mann-Whitney *U*-tests to identify domains enriched in high-PCN plasmids. To account for multiple comparisons, we applied Benjamini-Hochberg false discovery rate (FDR) correction, with statistical significance defined as FDR-adjusted *p* < 0.05. Enriched domains were further filtered to include only those showing both statistical significance (FDR *p* < 0.05) and biological relevance (fold-enrichment > 1 in high-PCN plasmids). For visualization, we focused on the top 20 most enriched domains (ranked by raw domain frequency) to highlight the strongest associations with high copy number maintenance.

### Reporting summary

Further information on research design is available in the Nature Portfolio Reporting Summary linked to this article.

## Supplementary information

Supplementary Information

Peer Review file

Reporting Summary

## Source data

Source Data

## Data Availability

All the data associated with this work are available at the GitHub repository (https://github.com/Iqra123isynbio/Plasmid_copy_number_Prediction) and have been archived on Zenodo 10.5281/zenodo.19343249^65^. Source data are provided with this paper.

## Code availability

All codes are available at the Github repository (https://github.com/Iqra123isynbio/Plasmid_copy_number_Prediction) and have been archived on Zenodo 10.5281/zenodo.19343249^65^.

## References

[1] Rodríguez-Beltrán, J., DelaFuente, J., León-Sampedro, R., MacLean, R. C. & San Millán, Á Beyond horizontal gene transfer: the role of plasmids in bacterial evolution. *Nat. Rev. Microbiol.* **19**, 347–359 (2021). 33469168 10.1038/s41579-020-00497-1

[2] Ramiro-Martínez, P., de Quinto, I., Lanza, V. F., Gama, J. A. & Rodríguez-Beltrán, J. Universal rules govern plasmid copy number. *Nat. Commun.* **16**, 6022 (2025). 40603299 10.1038/s41467-025-61202-5 PMC12223202

[3] Maddamsetti, R. et al. Scaling laws of bacterial and archaeal plasmids. *Nat. Commun.* **16**, 6023 (2025). 40603865 10.1038/s41467-025-61205-2 PMC12222811

[4] San Millan, A., Escudero, J. A., Gifford, D. R., Mazel, D. & MacLean, R. C. Multicopy plasmids potentiate the evolution of antibiotic resistance in bacteria. *Nat. Ecol. Evol.* **1**, 0010 (2016). 10.1038/s41559-016-0010 28812563

[5] Yao, Y. et al. Intra-and interpopulation transposition of mobile genetic elements driven by antibiotic selection. *Nat. Ecol. Evol.* **6**, 555–564 (2022). 35347261 10.1038/s41559-022-01705-2 PMC12520065

[6] Hernandez-Beltran, J. C. R. et al. Plasmid-mediated phenotypic noise leads to transient antibiotic resistance in bacteria. *Nat. Commun.* **15**, 2610 (2024). 38521779 10.1038/s41467-024-45045-0 PMC10960800

[7] Wang, H. et al. Increased plasmid copy number is essential for Yersinia T3SS function and virulence. *Science* **353**, 492–495 (2016). 27365311 10.1126/science.aaf7501

[8] Sidhu, R. K. et al. Attenuation of virulence in Yersinia pestis across three plague pandemics. *Science* **388**, eadt3880 (2025). 40440389 10.1126/science.adt3880

[9] Wang, H. & Joffré, E. Plasmid copy number as a modulator in bacterial pathogenesis and antibiotic resistance. *npj Antimicrob. Resist.* **3**, 72 (2025). 40826263 10.1038/s44259-025-00145-9 PMC12361487

[10] Joshi, S. H.-N., Yong, C. & Gyorgy, A. Inducible plasmid copy number control for synthetic biology in commonly used E. coli strains. *Nat. Commun.* **13**, 6691 (2022). 36335103 10.1038/s41467-022-34390-7 PMC9637173

[11] Kumar, S., Lezia, A. & Hasty, J. Engineering plasmid copy number heterogeneity for dynamic microbial adaptation. *Nat. Microbiol.* **9**, 2173–2184 (2024). 38890490 10.1038/s41564-024-01706-w PMC11623956

[12] Rouches, M. V., Xu, Y., Cortes, L. B. G. & Lambert, G. A plasmid system with tunable copy number. *Nat. Commun.* **13**, 3908 (2022). 35798738 10.1038/s41467-022-31422-0 PMC9263177

[13] Son, H. -I. et al. Population-level amplification of gene regulation by programmable gene transfer. *Nat. Chem. Biol.* **21**, 939–948 (2025). 39779901 10.1038/s41589-024-01817-9 PMC12599128

[14] Camargo, A. P. et al. IMG/PR: a database of plasmids from genomes and metagenomes with rich annotations and metadata. *Nucleic Acids Res.* **52**, D164–D173 (2024). 37930866 10.1093/nar/gkad964 PMC10767988

[15] Lee, C., Kim, J., Shin, S. G. & Hwang, S. Absolute and relative QPCR quantification of plasmid copy number in Escherichia coli. *J. Biotechnol.* **123**, 273–280 (2006). 16388869 10.1016/j.jbiotec.2005.11.014

[16] Browne, H. P. et al. Culturing of ‘unculturable’ human microbiota reveals novel taxa and extensive sporulation. *Nature* **533**, 543–546 (2016). 27144353 10.1038/nature17645 PMC4890681

[17] Zhang, Z. et al. Assessment of global health risk of antibiotic resistance genes. *Nat. Commun.* **13**, 1553 (2022). 35322038 10.1038/s41467-022-29283-8 PMC8943045

[18] Lucks, J. B., Qi, L., Whitaker, W. R. & Arkin, A. P. Toward scalable parts families for predictable design of biological circuits. *Curr. Opin. Microbiol.* **11**, 567–573 (2008). 18983935 10.1016/j.mib.2008.10.002

[19] Leinonen, R., Sugawara, H., Shumway, M. & Collaboration, I. N. S. D. The sequence read archive. *Nucleic Acids Res.* **39**, D19–D21 (2010). 21062823 10.1093/nar/gkq1019 PMC3013647

[20] Soppa, J. Polyploidy and community structure. *Nat. Microbiol.* **2**, 1–2 (2017). 10.1038/nmicrobiol.2016.261 28120929

[21] Mendell, J. E., Clements, K. D., Choat, J. H. & Angert, E. R. Extreme polyploidy in a large bacterium. *Proc. Natl. Acad. Sci.* **105**, 6730–6734 (2008). 18445653 10.1073/pnas.0707522105 PMC2373351

[22] Takacs, C. N. et al. Polyploidy, regular patterning of genome copies, and unusual control of DNA partitioning in the Lyme disease spirochete. *Nat. Commun.* **13**, 7173 (2022). 36450725 10.1038/s41467-022-34876-4 PMC9712426

[23] Hamrick, G. S. et al. Programming dynamic division of labor using horizontal gene transfer. *ACS Synth. Biol.* **13**, 1142–1151 (2024). 38568420 10.1021/acssynbio.3c00615 PMC12519958

[24] Xue, W., Hong, J. & Wang, T. The evolutionary landscape of prokaryotic chromosome/plasmid balance. *Commun. Biol.* **7**, 1434 (2024). 39496780 10.1038/s42003-024-07167-5 PMC11535066

[25] Doolittle, W. F. & Sapienza, C. Selfish genes, the phenotype paradigm and genome evolution. *Nature* **284**, 601–603 (1980). 6245369 10.1038/284601a0

[26] Lopatkin, A. J. et al. Persistence and reversal of plasmid-mediated antibiotic resistance. *Nat. Commun.* **8**, 1689 (2017). 29162798 10.1038/s41467-017-01532-1 PMC5698434

[27] Wang, T. & You, L. The persistence potential of transferable plasmids. *Nat. Commun.* **11**, 5589 (2020). 33149119 10.1038/s41467-020-19368-7 PMC7642394

[28] Hall, J. P. et al. Plasmid fitness costs are caused by specific genetic conflicts enabling resolution by compensatory mutation. *PLos Biol.* **19**, e3001225 (2021). 34644303 10.1371/journal.pbio.3001225 PMC8544851

[29] Thomas, C. M. et al. Annotation of plasmid genes. *Plasmid* **91**, 61–67 (2017). 28365184 10.1016/j.plasmid.2017.03.006

[30] Yu, M. K., Fogarty, E. C. & Eren, A. M. Diverse plasmid systems and their ecology across human gut metagenomes revealed by PlasX and MobMess. *Nat. Microbiol.* **9**, 830–847 (2024). 38443576 10.1038/s41564-024-01610-3 PMC10914615

[31] Paysan-Lafosse, T. et al. The Pfam protein families database: embracing AI/ML. *Nucleic Acids Res.* **53**, D523–D534 (2025). 39540428 10.1093/nar/gkae997 PMC11701544

[32] Hyatt, D. et al. Prodigal: prokaryotic gene recognition and translation initiation site identification. *BMC Bioinforma.* **11**, 119 (2010). 10.1186/1471-2105-11-119 PMC2848648 20211023

[33] Finn, R. D. et al. Pfam: clans, web tools and services. *Nucleic Acids Res.* **34**, D247–D251 (2006). 16381856 10.1093/nar/gkj149 PMC1347511

[34] Motallebi-Veshareh, M., Rouch, D. & Thomas, C. A family of ATPases involved in active partitioning of diverse bacterial plasmids. *Mol. Microbiol.* **4**, 1455–1463 (1990). 2149583 10.1111/j.1365-2958.1990.tb02056.x

[35] Mitchell, A. et al. The InterPro protein families database: the classification resource after 15 years. *Nucleic Acids Res.* **43**, D213–D221 (2015). 25428371 10.1093/nar/gku1243 PMC4383996

[36] Campbell, E. A. et al. Structure of the bacterial RNA polymerase promoter specificity σ subunit. *Mol. Cell* **9**, 527–539 (2002). 11931761 10.1016/s1097-2765(02)00470-7

[37] Seabold, R. R. & Schleif, R. F. Apo-AraC actively seeks to loop. *J. Mol. Biol.* **278**, 529–538 (1998). 9600836 10.1006/jmbi.1998.1713

[38] Kleiger, G. & Eisenberg, D. GXXXG and GXXXA motifs stabilize FAD and NAD (P)-binding Rossmann folds through Cα–H⋯ O hydrogen bonds and van der Waals interactions. *J. Mol. Biol.* **323**, 69–76 (2002). 12368099 10.1016/s0022-2836(02)00885-9

[39] De Meo, P., Ferrara, E., Fiumara, G. & Provetti, A. Generalized louvain method for community detection in large networks. *11th international conference on intelligent systems design and applications*, 88-93 (2011).

[40] Bethke, J. H. et al. Environmental and genetic determinants of plasmid mobility in pathogenic Escherichia coli. *Sci. Adv.* **6**, eaax3173 (2020). 32042895 10.1126/sciadv.aax3173 PMC6981087

[41] Cooper, A. L., Wong, A., Tamber, S., Blais, B. W. & Carrillo, C. D. Analysis of antimicrobial resistance in bacterial pathogens recovered from food and human sources: insights from 639,087 bacterial whole-genome sequences in the NCBI Pathogen Detection database. *Microorganisms* **12**, 709 (2024). 38674654 10.3390/microorganisms12040709 PMC11051753

[42] Alcock, B. P. et al. CARD 2023: expanded curation, support for machine learning, and resistome prediction at the Comprehensive Antibiotic Resistance Database. *Nucleic Aacids Res.* **51**, D690–D699 (2023). 10.1093/nar/gkac920 PMC9825576 36263822

[43] Avire, N. J., Whiley, H. & Ross, K. A review of Streptococcus pyogenes: public health risk factors, prevention and control. *Pathogens* **10**, 248 (2021). 33671684 10.3390/pathogens10020248 PMC7926438

[44] Higgins, D. A. et al. The major Vibrio cholerae autoinducer and its role in virulence factor production. *Nature* **450**, 883–886 (2007). 18004304 10.1038/nature06284

[45] Hazen, T. C., Fliermans, C. B., Hirsch, R. P. & Esch, G. W. Prevalence and distribution of Aeromonas hydrophila in the United States. *Appl. Environ. Microbiol.* **36**, 731–738 (1978). 31839 10.1128/aem.36.5.731-738.1978 PMC243130

[46] Salyers, A. A., Gupta, A. & Wang, Y. Human intestinal bacteria as reservoirs for antibiotic resistance genes. *Trends Microbiol.* **12**, 412–416 (2004). 15337162 10.1016/j.tim.2004.07.004

[47] Jurėnas, D., Fraikin, N., Goormaghtigh, F. & Van Melderen, L. Biology and evolution of bacterial toxin–antitoxin systems. *Nat. Rev. Microbiol.* **20**, 335–350 (2022). 34975154 10.1038/s41579-021-00661-1

[48] Luo, X. et al. Characterization of DinJ-YafQ toxin–antitoxin module in Tetragenococcus halophilus: activity, interplay, and evolution. *Appl. Microbiol. Biotechnol.* **105**, 3659–3672 (2021). 33877415 10.1007/s00253-021-11297-9

[49] San Millan, A. et al. Small-plasmid-mediated antibiotic resistance is enhanced by increases in plasmid copy number and bacterial fitness. *Antimicrob. Agents Chemother.* **59**, 3335–3341 (2015). 25824216 10.1128/AAC.00235-15 PMC4432117

[50] van Mastrigt, O., Lommers, M. M., de Vries, Y. C., Abee, T. & Smid, E. J. Dynamics in copy numbers of five plasmids of a dairy Lactococcus lactis strain under dairy-related conditions, including near-zero growth rates. *Appl. Environ. Microbiol.* **84**, e00314–00318 (2018). 29572209 10.1128/AEM.00314-18 PMC5960958

[51] Uhlin, B. E., Molin, S., Gustafsson, P. & Nordström, K. Plasmids with temperature-dependent copy number for amplification of cloned genes and their products. *Gene* **6**, 91–106 (1979). 383579 10.1016/0378-1119(79)90065-9

[52] Akasaka, N. et al. Change in the plasmid copy number in acetic acid bacteria in response to growth phase and acetic acid concentration. *J. Biosci. Bioeng.* **119**, 661–668 (2015). 25575969 10.1016/j.jbiosc.2014.11.003

[53] Saad, A. et al. Plasmid copy number variation impacts pathogenicity and quantification of Curtobacterium flaccumfaciens pv. flaccumfaciens infecting the mung bean. *Plant Pathol.* **74**, 2670–2681 (2025).

[54] Wedel, E. et al. Insertion sequences determine plasmid adaptation to new bacterial hosts. *MBio* **14**, e03158–03122 (2023). 37097157 10.1128/mbio.03158-22 PMC10294622

[55] Peng, K. et al. Long-read metagenomic sequencing reveals that high-copy small plasmids shape the highly prevalent antibiotic resistance genes in the animal fecal microbiome. *Sci. Total Environ.* **893**, 164585 (2023). 37269991 10.1016/j.scitotenv.2023.164585

[56] Potter, S. C. et al. HMMER web server: 2018 update. *Nucleic acids Res.* **46**, W200–W204 (2018). 29905871 10.1093/nar/gky448 PMC6030962

[57] Ahlmann-Eltze, C. & Huber, W. Comparison of transformations for single-cell RNA-seq data. *Nat. Methods* **20**, 665–672 (2023). 37037999 10.1038/s41592-023-01814-1 PMC10172138

[58] Pedregosa, F. *et al*. *Scikit-learn: Machine Learning in Python.* (2012).

[59] Chen, T. & Guestrin, C. Xgboost: A scalable tree boosting system. *Proceedin**gs of the 22nd ACM SIGKDD International Conference on Knowledge Discovery and Data Mining*. 785–794 (2016).

[60] Rajewska, M., Wegrzyn, K. & Konieczny, I. AT-rich region and repeated sequences – the essential elements of replication origins of bacterial replicons. *FEMS Microbiol. Rev.* **36**, 408–434 (2012). 22092310 10.1111/j.1574-6976.2011.00300.x

[61] Shannon, P. et al. Cytoscape: a software environment for integrated models of biomolecular interaction networks. *Genome Res.* **13**, 2498–2504 (2003). 14597658 10.1101/gr.1239303 PMC403769

[62] Traag, V. A., Waltman, L. & van Eck, N. J. From Louvain to Leiden: guaranteeing well-connected communities. *Sci. Rep.* **9**, 5233 (2019). 30914743 10.1038/s41598-019-41695-z PMC6435756

[63] Robertson, J. ames & Nash John H, E. MOB-suite: software tools for clustering, reconstruction and typing of plasmids from draft assemblies. *Microb. Genom.* **4**, e000206 (2018). 30052170 10.1099/mgen.0.000206 PMC6159552

[64] Seemann, T. Prokka: rapid prokaryotic genome annotation. *Bioinformatics* **30**, 2068–2069 (2014). 24642063 10.1093/bioinformatics/btu153

[65] Shahzadi, I. *et al*. Integrating theory and machine learning to reveal determinants of plasmid copy number. *Github*10.5281/zenodo.19343249 (2026). 10.1038/s41467-026-72303-0 PMC13287686 42020421

