# Supplementary material for: Integrating theory and machine learning to reveal determinants of plasmid copy number
Source: Nat Commun. 2026 Apr 22;17:5539. doi: 10.1038/s41467-026-72303-0 (PMC13287686; doi:10.1038/s41467-026-72303-0)
Supplement: Supplementary file 1 — Supplementary Information [file 41467_2026_72303_MOESM1_ESM.pdf]

Supplementary Information for

Integrating theory and machine learning to reveal determinants of plasmid copy number

Iqra Shahzadi[1,2], Wenzhi Xue[1], Hasan Ubaid Ullah[2], Rohan Maddamsetti[3], Lingchong You[4,5,6], Teng Wang[1]✉

[1]State Key Laboratory of Quantitative Synthetic Biology, Shenzhen Institute of Synthetic Biology, Shenzhen Institutes of Advanced Technology, Chinese Academy of Sciences, Shenzhen, China

[2]University of Chinese Academy of Sciences, Beijing, China

[3]Department of Biochemistry and Microbiology, Rutgers University, New Brunswick, NJ, USA

[4]Center for Quantitative Biodesign, Duke University, Durham, NC, USA

[5]Department of Biomedical Engineering, Duke University, Durham, NC, USA

[6]Department of Molecular Genetics and Microbiology, Duke University School of Medicine, Durham, NC, USA

✉Correspondence: t.wang1@siat.ac.cn

Supplementary Text

A simple model explaining the power law relationship between plasmid size and copy number

To explain the power-law relationship between plasmid size and copy number, we adapted a model describing the intracellular resource competition between the chromosome and plasmids[1]. In this framework, the strengths of host-level and plasmid-level selections are determined by how resources are distributed between the chromosome and plasmids.

Let C, P and θ denote chromosome size, plasmid size and PCN, respectively. Both C and P are assumed to be proportional to the number of genes encoded[1]. We further assume that a larger gene repertoire enhanced the cell's ability to exploit diverse environmental resources, thereby increasing the total intracellular resource availability R_{total}. The relationship between genome size and R_{total} is described by a Hill function:

$$R_{total} = \frac{M(C + h\theta P)}{K + C + h\theta P}. \quad (1)$$

Here, M is the maximum attainable resource availability, h is a constant reflecting the relative advantage of plasmid genes in resource competition. K is the value of $C + h\theta P$ at which half of M is reached.

The resources devoted to plasmid functions (R_P) are determined by the ratio of plasmid DNA to total DNA:

$$\frac{R_P}{R_{total}} = \frac{h\theta P}{C + h\theta P}. \quad (2)$$

Combining equation (1) and (2) gives:

$$R_P = \frac{h\theta M P}{K + C + h\theta P}. \quad (3)$$

Plasmid-level selection favors greater $\frac{R_P}{R_{total}}$. Therefore, we defined the cost function ($\phi_{plasmid}$) of plasmid-level selection as:

$$\phi_{plasmid} = 1 - \frac{R_P}{R_{total}} = \frac{C}{C + h\theta P}. \quad (4)$$

$\phi_{plasmid}$ can be reduced by increasing θ or P.

Similarly, the resources allocated to host functions (R_C, encoded by chromosome) are:

$$R_C = \frac{MC}{K + C + h\theta P}. \quad (5)$$

When the host is plasmid-free, all resources are allocated to the chromosome, giving:

$$R_0 = \frac{MC}{K + C}. \quad (6)$$

Plasmid carriage reduces this allocation to R_C, and the different between R_C and R_0 represents the metabolic burden of plasmid maintenance. We therefore define the cost function of host-level selection as:

$$\phi_{host} = 1 - \frac{R_C}{R_0}, \quad (7)$$

which simplifies to:

$$\phi_{host} = \frac{h\theta P}{K + C + h\theta P}. \quad (8)$$

Host-level selection thus acts to minimize ϕ_{host} by reducing θ or P.

The total cost function is given by the sum of the two components:

$$\phi_{total} = \frac{h\theta P}{K + C + h\theta P} + \frac{C}{C + h\theta P}. \quad (9)$$

Here, PCN (θ) increases ϕ_{host} but decreases $\phi_{plasmid}$. Consequently, equation (9) predicts the biphasic change of ϕ_{total} with increasing θ: starting from 1, ϕ_{total} decreases to a minimum, then rises back toward 1. At low θ, $\phi_{plasmid}$ dominates; at high θ, ϕ_{host} dominates. The minimum of ϕ_{total} represents the evolutionary optimum of θ.

To obtain this optimum analytically, we calculate the derivative of ϕ_{total} with respect to θ:

$$\frac{\partial \phi_{total}}{\partial \theta} = \frac{hKP + hPC}{(K + C + h\theta P)^2} - \frac{hPC}{(C + h\theta P)^2}. \quad (10)$$

The optimal θ, denoted as θ^*, could be derived by solving the equation $\frac{\partial \phi_{total}}{\partial \theta} = 0$:

$$\frac{hKP + hPC}{(K + C + h\theta P)^2} - \frac{hPC}{(C + h\theta P)^2} = 0, \quad (11)$$

which led to

$$\theta^* = \frac{\sqrt{KC + C^2}}{hP}. \quad (12)$$

Equation (12) reveals the power-law relationship between optimal PCN (θ^*) and plasmid size (P). Moreover, it predicts that the total plasmid DNA content ($\theta^* \cdot P$) scales positively with chromosome size (C):

$$\theta^* \cdot P = \frac{\sqrt{KC + C^2}}{h}. \quad (13)$$

This prediction is consistent with observations from our PCN dataset.

Supplementary Figures

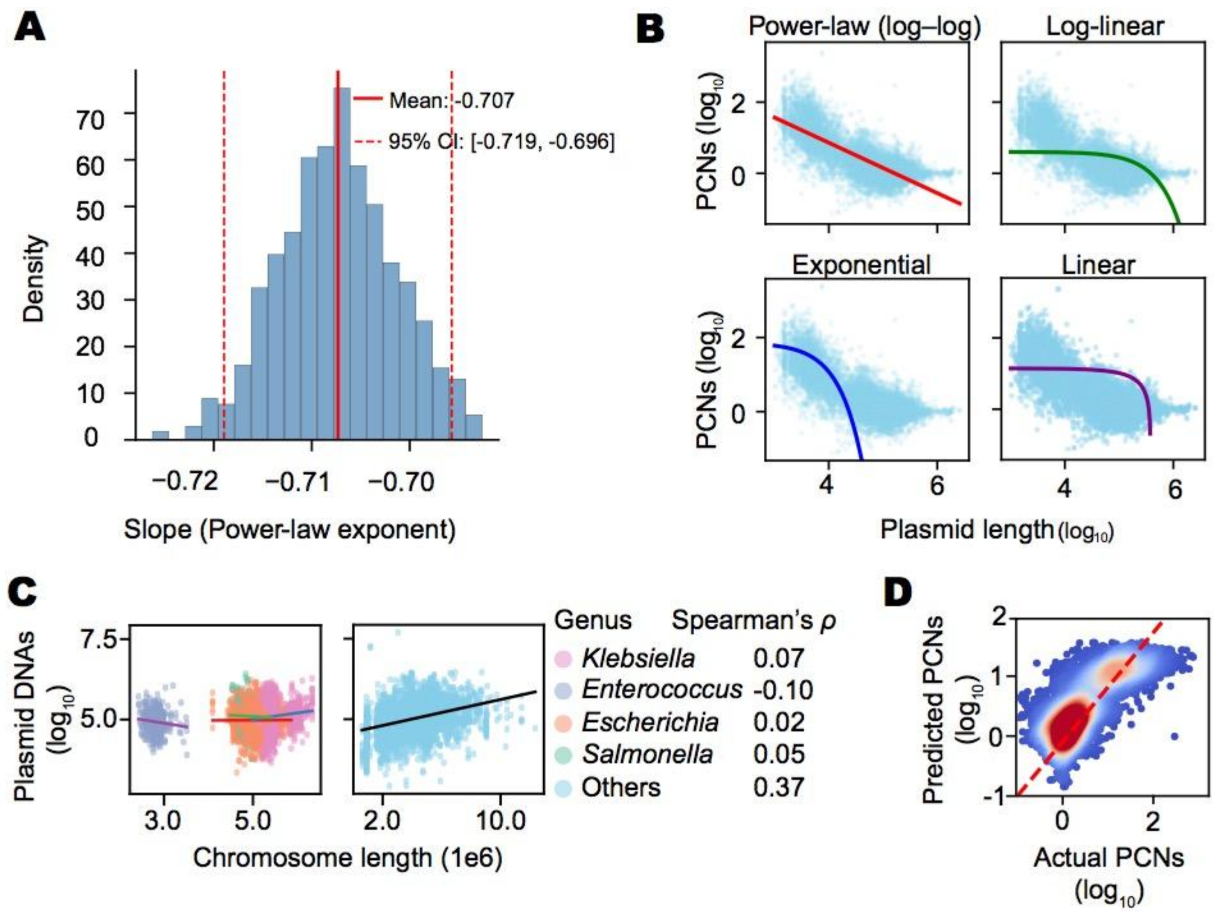

Figure S1| Plasmid size alone shows limited predictive power for PCN.

(A) Bootstrap distribution of the power-law scaling exponent estimated from 1,000 resampling iterations. The red solid line indicates the mean exponent (–0.707), and the red dashed lines mark the 95% confidence interval (–0.719 to –0.696), confirming the stability of the estimate.

(B) Comparison of alternative functional forms for the plasmid size-PCN relationship. Power-law (log–log), log-linear, exponential, and linear models were evaluated using Akaike Information Criterion (AIC). The power-law model shows superior fit (ΔAIC > 9,000) relative to next-best alternative.

(C) Correlation between plasmid DNA amounts and chromosome size. Data for the four most prevalent bacterial genera (*Escherichia, Klebsiella*, *Salmonella*, and *Enterococcus*) are shown on the left, while other genera are displayed on the right. Log10-transformed plasmid DNA amounts are plotted against chromosome size, with linear regression lines fitted to each group.

(D) Correlation (R^2 ~0.63) between actual PCNs and predicted PCNs derived from the power-law relationship between plasmid size and PCN. Point density is represented by color shading.

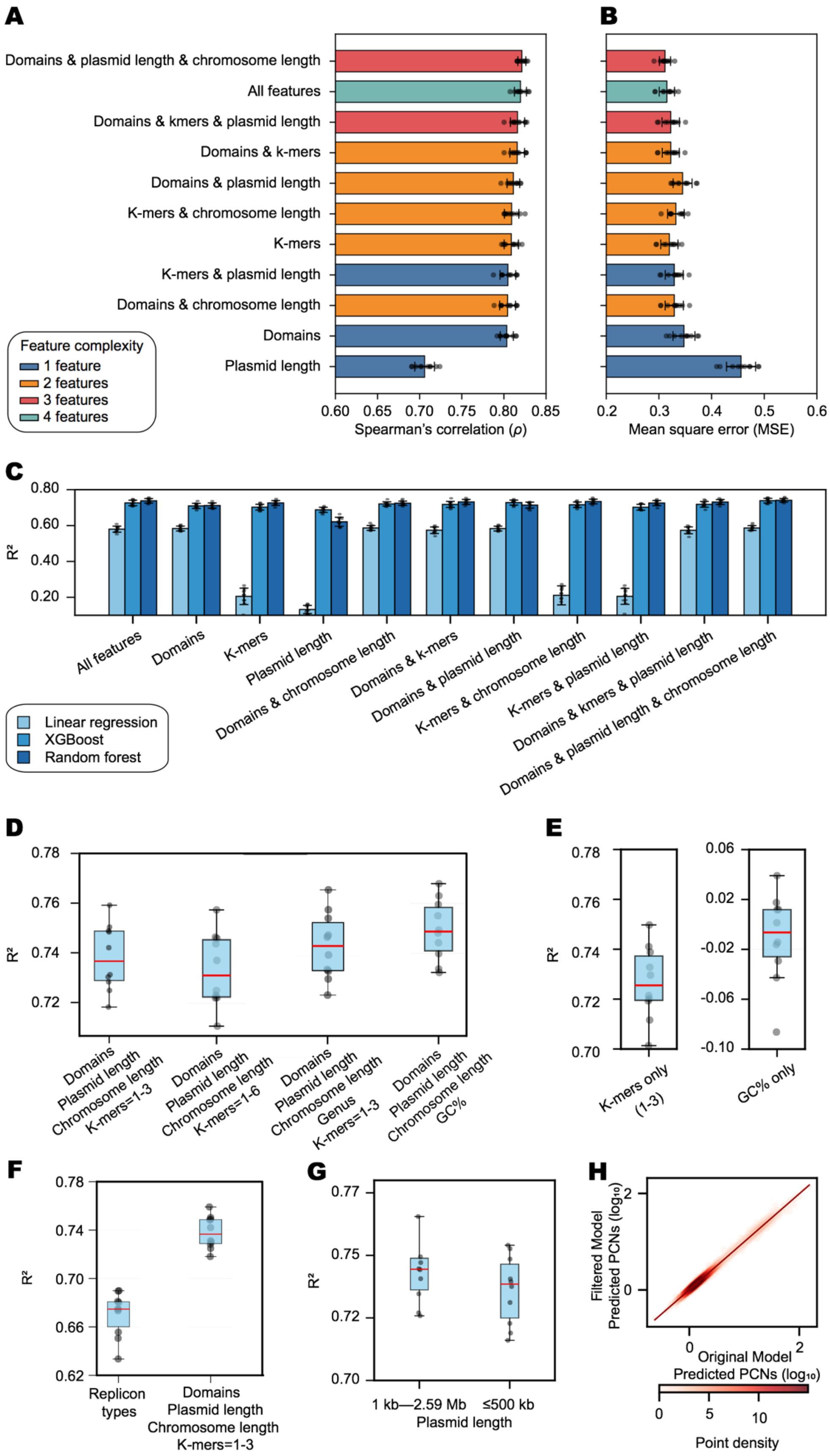

A
Domains & plasmid length & chromosome length
All features
Domains & kmers & plasmid length
Domains & k-mers
Domains & plasmid length
K-mers & chromosome length
K-mers
K-mers & plasmid length
Domains & chromosome length
Domains
Plasmid length
Feature complexity
1 feature
2 features
3 features
4 features
Spearman's correlation (ρ)
B
Mean square error (MSE)
C
R²
All features
Domains
K-mers
Plasmid length
Domains & chromosome length
Domains & k-mers
Domains & plasmid length
K-mers & chromosome length
K-mers & plasmid length
Domains & kmers & plasmid length
Domains & plasmid length & chromosome length
Linear regression
XGBoost
Random forest
D
Domains Plasmid length Chromosome length K-mers=1-3
Domains Plasmid length Chromosome length K-mers=1-6
Domains Plasmid length Chromosome length Genus K-mers=1-3
Domains Plasmid length Chromosome length GC%
E
K-mers only (1-3)
GC% only
F
Replicon types
Domains Plasmid length Chromosome length K-mers=1-3
G
1 kb—2.59 Mb
≤500 kb
Plasmid length
H
Filtered Model Predicted PCNs (log₁₀)
Original Model Predicted PCNs (log₁₀)
Point density

Figure S2| The predictive performances of the modeling frameworks using different sequence-derived features.

(A) Model performances evaluated by the Spearman's ρ between actual and predicted PCNs. Data were presented as mean ± standard deviation across 10 replicates.

(B) Model performances evaluated by MSE (mean squared error) between actual and predicted PCNs. Data were presented as mean ± standard deviation across 10 replicates.

(C) Comparison of regression algorithms across feature combinations. The random forest regressor consistently outperformed linear regression and XGBoost across all tested feature sets, indicating its superior capacity to model non-linear relationships between molecular features and PCN. Data were presented as mean ± standard deviation across 10 replicates.

(D) Model performance across different feature sets evaluated via 10-fold cross-validation. Boxplots show R^2 distributions for models trained with: all features with host genus (k=1-3), all features with varying k-mer lengths (k=1-6), all features with GC%, k-mers alone (1-3), and GC% alone. Host genus and GC% provided marginal improvements over core molecular features.

(E) When used alone to predict PCNs, *k*-mers substantially outperformed GC%. Boxplots show R^2 distributions of 10 replicates.

(F) Performance comparison of the domain-centric predictive framework versus replicon-type classification. Our model significantly outperforms prediction based solely on replicon type, demonstrating that molecular features provide predictive power beyond broad plasmid family classification. Boxplots show R^2 distributions of 10 replicates.

(G) Sensitivity analysis on plasmid size gating. Model performance is compared between the full dataset (1 kb to 2.59 MB; n=11,051) and a filtered dataset excluding large plasmids (≤500 kb; n=10,862). Boxplots show R^2 distributions from 10 replicates, demonstrating stable performance ($\Delta R^2 = 0.010$) after removal of megaplasmids or potential secondary chromosomes.

(H) The PCN predictions for the overlapping plasmid set between the two models (trained on full dataset or filtered dataset excluding large plasmids) were strongly correlated (Pearson's r = 0.94).

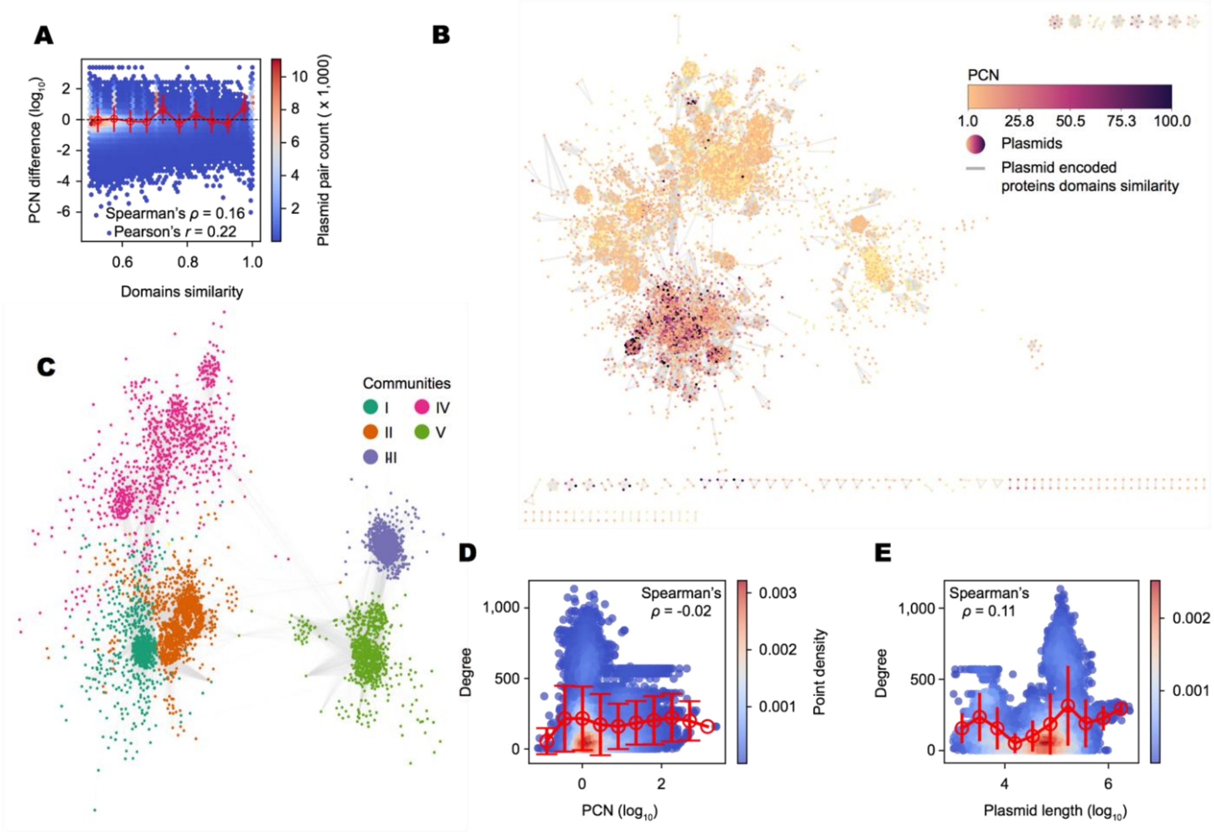

Figure S3| Plasmid similarity network based on their encoded protein domains.

(A) Relationship between cosine similarity scores and PCN differences across all plasmid pairs. For each pair, PCN difference is defined as the ratio of the larger PCN to the smaller one. Bar plots represent binned averages ± standard deviations.

(B) Network visualization. Nodes represent individual plasmids, colored by their PCNs. Edges connect plasmids with a cosine similarity of ⩾ 0.7 in their protein domain compositions.

(C) Community structure of the plasmid network. Nodes are colored according to their assigned community membership (I-V).

(D) Association between plasmid degree (connectivity) and PCN. PCN values were grouped into equal-width bins, with bar plots showing the mean degree ± standard deviation within each bin. Degree is defined as the number of plasmids connected to a given plasmid in the network created with the threshold of cosine similarity ⩾ 0.5.

(E) Relationship between plasmid degree and plasmid size.

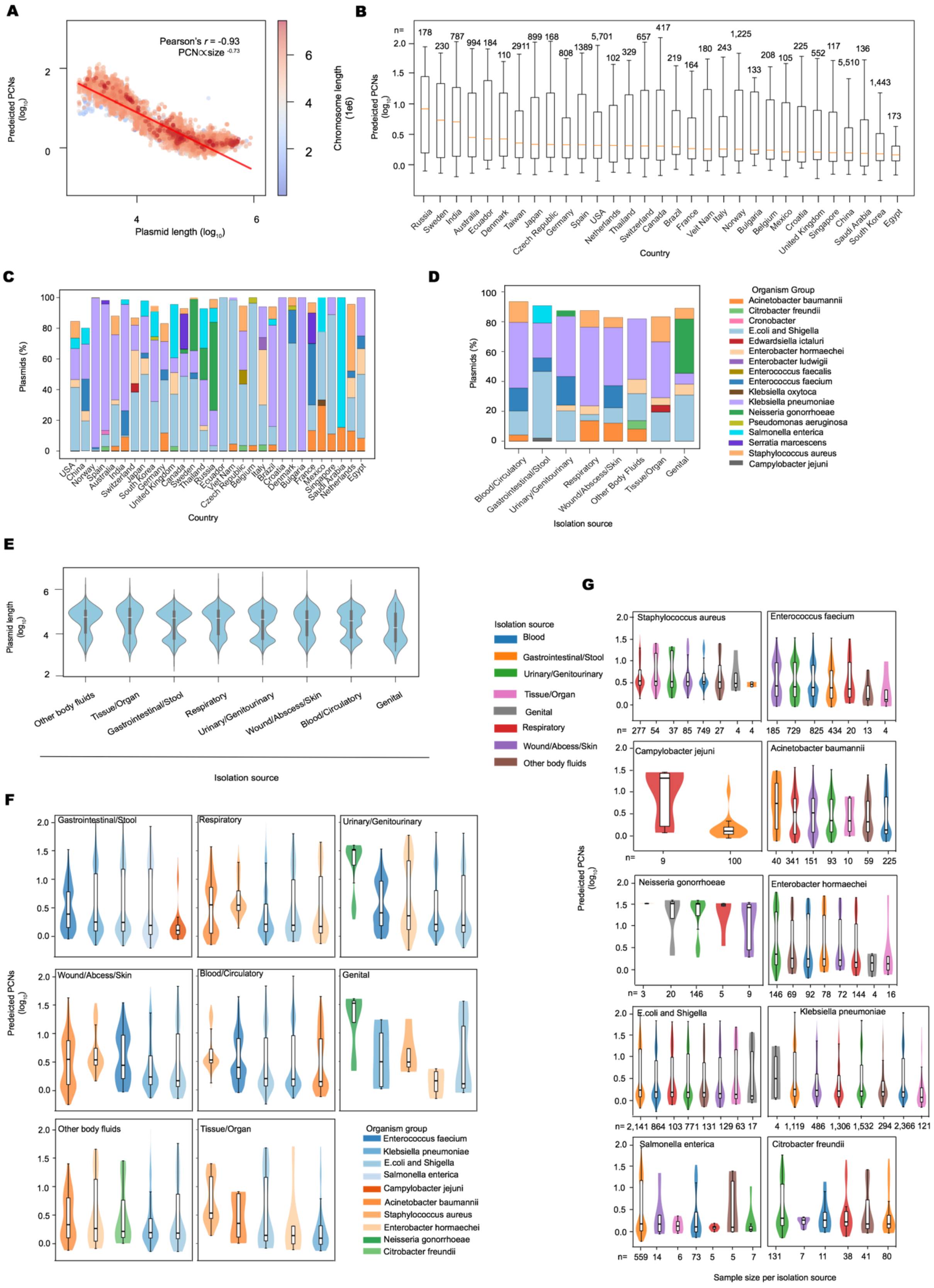
A
Pearson's r = -0.93
PCN∝size -0.73
Predeicted PCNs (log10)
Plasmid length (log10)
Chromosome length (1e6)
B
n=
Predeicted PCNs (log10)
Country
Russia
Sweden
India
Australia
Ecuador
Denmark
Taiwan
Japan
Czech Republic
Germany
Spain
USA
Netherlands
Thailand
Switzerland
Canada
Brazil
France
Veit Nam
Italy
Norway
Bulgaria
Belgium
Mexico
Croatia
United Kingdom
Singapore
China
Saudi Arabia
South Korea
Egypt
178
230
787
994
184
110
2911
899
168
808
1389
5,701
102
329
657
417
219
164
180
243
1,225
133
208
105
225
552
117
5,510
136
1,443
173
C
Plasmids (%)
Country
D
Plasmids (%)
Isolation source
Blood/Circulatory
Gastrointestinal/Stool
Urinary/Genitourinary
Respiratory
Wound/Abscess/Skin
Other Body Fluids
Tissue/Organ
Genital
Organism Group
Acinetobacter baumannii
Citrobacter freundii
Cronobacter
E.coli and Shigella
Edwardsiella ictaluri
Enterobacter hormaechei
Enterobacter ludwigii
Enterococcus faecalis
Enterococcus faecium
Klebsiella oxytoca
Klebsiella pneumoniae
Neisseria gonorrhoeae
Pseudomonas aeruginosa
Salmonella enterica
Serratia marcescens
Staphylococcus aureus
Campylobacter jejuni
E
Plasmid length (log10)
Other body fluids
Tissue/Organ
Gastrointestinal/Stool
Respiratory
Urinary/Genitourinary
Wound/Abscess/Skin
Blood/Circulatory
Genital
Isolation source
Isolation source
Blood
Gastrointestinal/Stool
Urinary/Genitourinary
Tissue/Organ
Genital
Respiratory
Wound/Abcess/Skin
Other body fluids
F
Predeicted PCNs (log10)
Gastrointestinal/Stool
Respiratory
Urinary/Genitourinary
Wound/Abcess/Skin
Blood/Circulatory
Genital
Other body fluids
Tissue/Organ
Organism group
Enterococcus faecium
Klebsiella pneumoniae
E.coli and Shigella
Salmonella enterica
Campylobacter jejuni
Acinetobacter baumannii
Staphylococcus aureus
Enterobacter hormaechei
Neisseria gonorrhoeae
Citrobacter freundii
G
Predeicted PCNs (log10)
Staphylococcus aureus
n= 277 54 37 85 749 27 4 4
Enterococcus faecium
185 729 825 434 20 13 4
Campylobacter jejuni
n= 9 100
Acinetobacter baumannii
40 341 151 93 10 59 225
Neisseria gonorrhoeae
n= 3 20 146 5 9
Enterobacter hormaechei
146 69 92 78 72 144 4 16
E.coli and Shigella
n= 2,141 864 103 771 131 129 63 17
Klebsiella pneumoniae
4 1,119 486 1,306 1,532 294 2,366 121
Salmonella enterica
n= 559 14 6 73 5 5 7
Citrobacter freundii
131 7 11 38 41 80
Sample size per isolation source

Figure S4| Distribution patterns of predicted PCNs in clinical plasmids.

(A) Power-law relationship between predicted PCNs and sizes of clinical plasmids, with a scaling coefficient of -0.73. Point density is indicated by color shading (coolwarm scale).

(B) The distribution of predicted PCNs across different countries. Here, n represents the number of clinical plasmids mapped to each country and is shown on the top of each box.

(C) Taxonomic composition across clinical niches, stratified by geographic region. Different organism groups are represented by distinct colors. The height of each colored bar represents the relative abundance.

(D) Taxonomic composition across clinical niches, stratified by body site.

(E) Distribution of plasmid size across various human isolation sources. Groups are ordered by median plasmid length (largest to smallest).

(F) Distribution of predicted PCNs across various organism groups, stratified by human isolation sources. Violin plots show the full distribution, with embedded box plots indicating the median and interquartile range (IQR). Median values are highlighted within each box plot, and groups are arranged in descending order of median PCN.

(G) Distribution of predicted PCNs across various human isolation sources, stratified by organism groups.

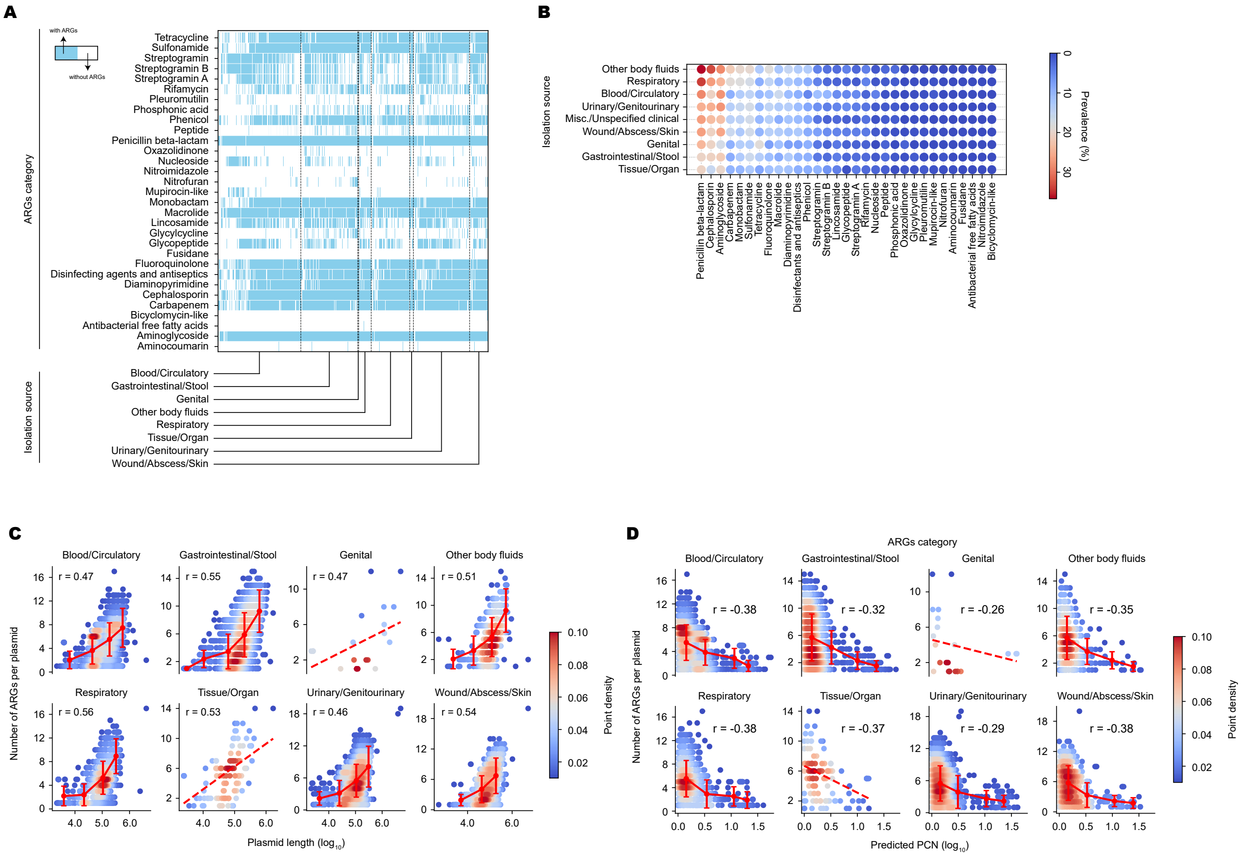

Figure S5| Distribution patterns of ARGs in clinical plasmids.

(A) Distributions of 32 categories of ARGs across plasmids in different human body systems.

(B) Prevalence of different ARG types in different human body systems.

(C) Relationship between plasmid length and the number of ARGs per plasmid across body systems. Point density is indicated by color shading, while bar plots show binned averages ± standard deviations. r denotes Pearson's correlation coefficient.

(D) Relationship between predicted PCN and the number of ARGs across body systems.

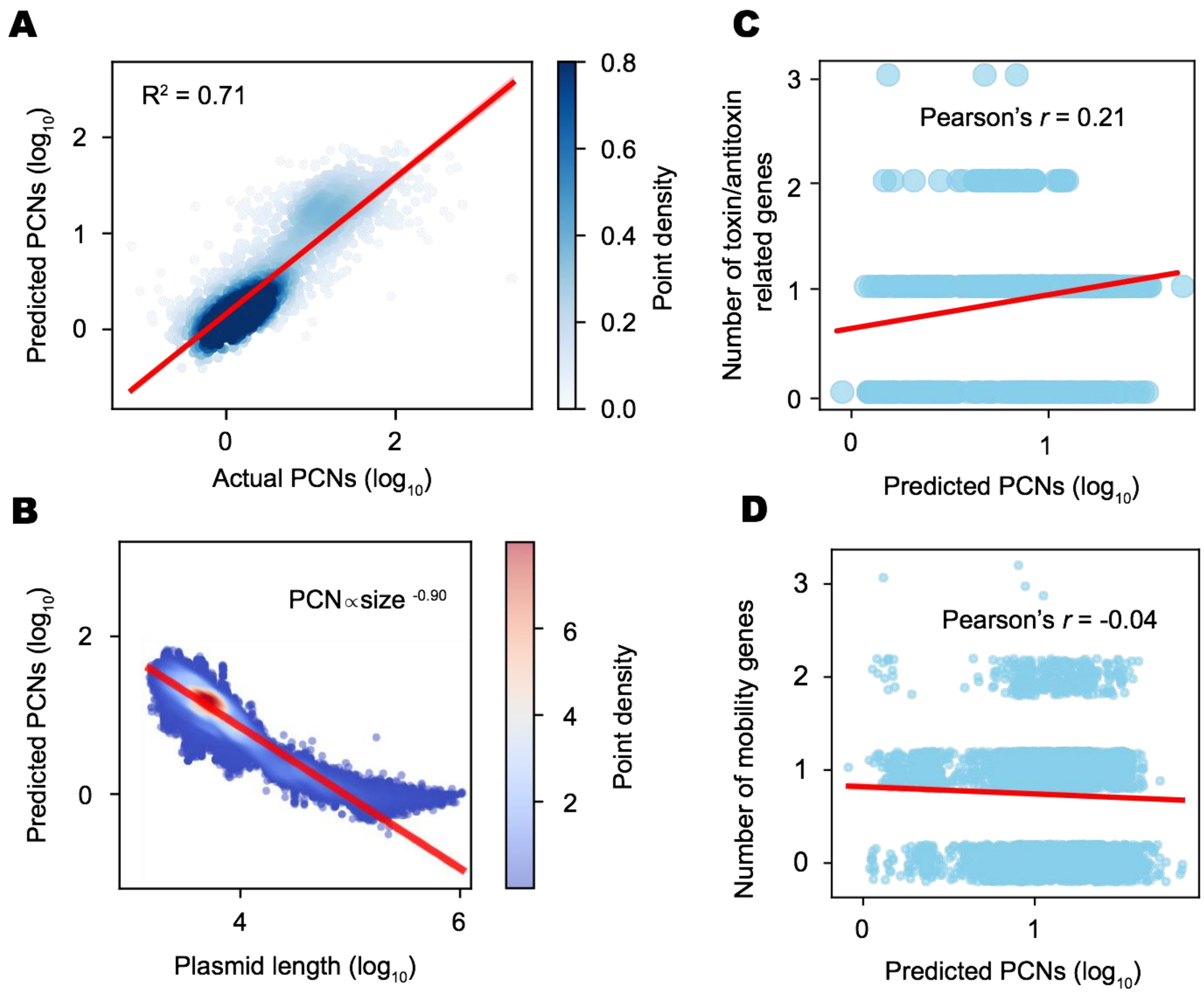

Figure S6| Distribution pattern of predicted PCNs across ecosystems.

(A) Correlation between predicted and actual PCNs in IMG/PR dataset. Point density is indicated by color shading (darker indicates higher density).

(B) Power-law relationship between plasmid size and predicted PCN in IMG/PR dataset, with a scaling coefficient of -0.90.

(C) Correlation between predicted PCNs and the number of toxin/antitoxin genes per human gut plasmid. The red line represents the linear regression.

(D) Relationship between predicted PCNs and the number of mobility-related genes per human gut plasmid.

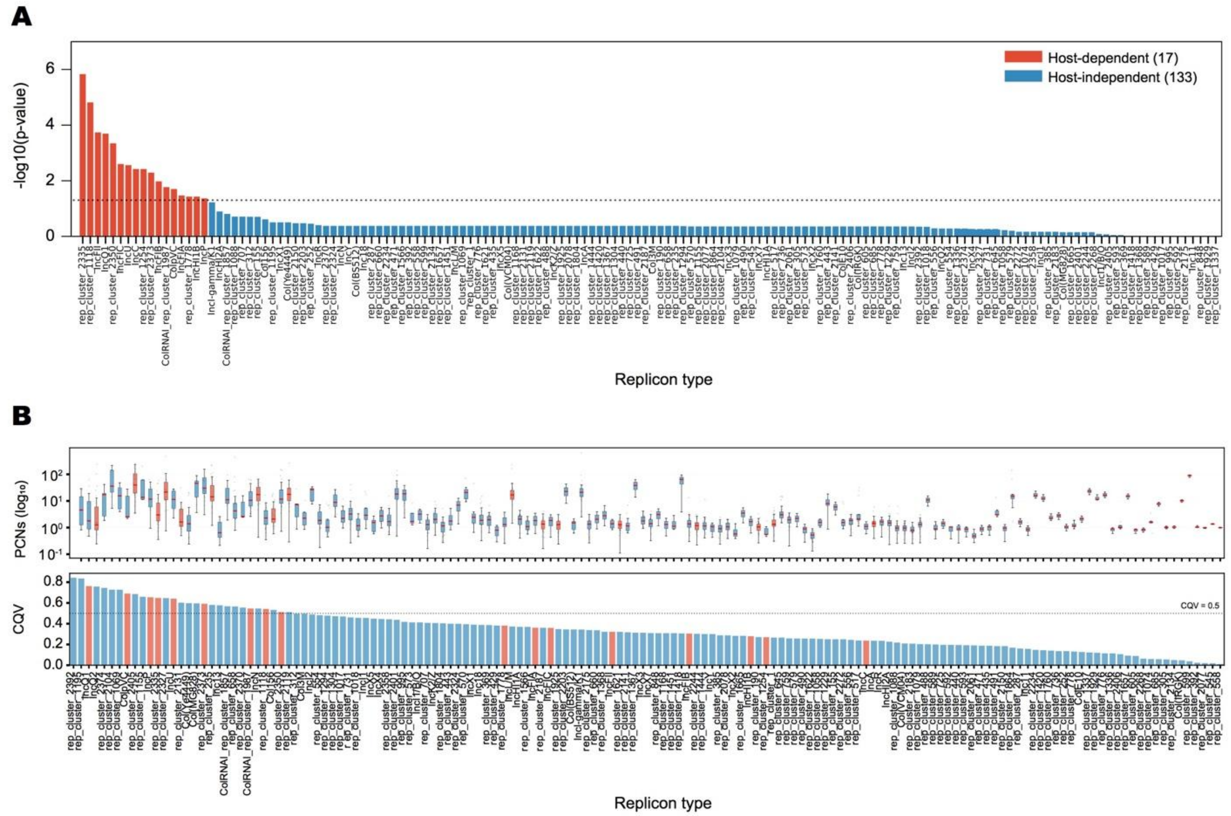

Figure S7| Host-independent PCN variation across replicon types.

(A) Assessment of host genus influence on PCN per replicon type. Each bar represents a replicon type, with bar height corresponding to the FDR-corrected p-value from a Kruskal-Wallis test for association between host genus and PCN. Replicon types with significant host-dependent PCN variation ($p \leqslant 0.05$) are colored red; those without significant variation ($p > 0.05$) are colored blue. The majority of replicon types (133 of 150, 88.7%) showed no significant host-dependent PCN variation.

(B) Characterization of internal PCN variability within replicon types. Top: Boxplots displaying the distribution of PCN values for replicon types, ordered by decreasing Coefficient of Quartile Variation (CQV). Bottom: Bar chart of the corresponding CQV values for each replicon type. Replicon types are colored according to their host-dependence classification from panel (A) (red: host-dependent; blue: host-independent).

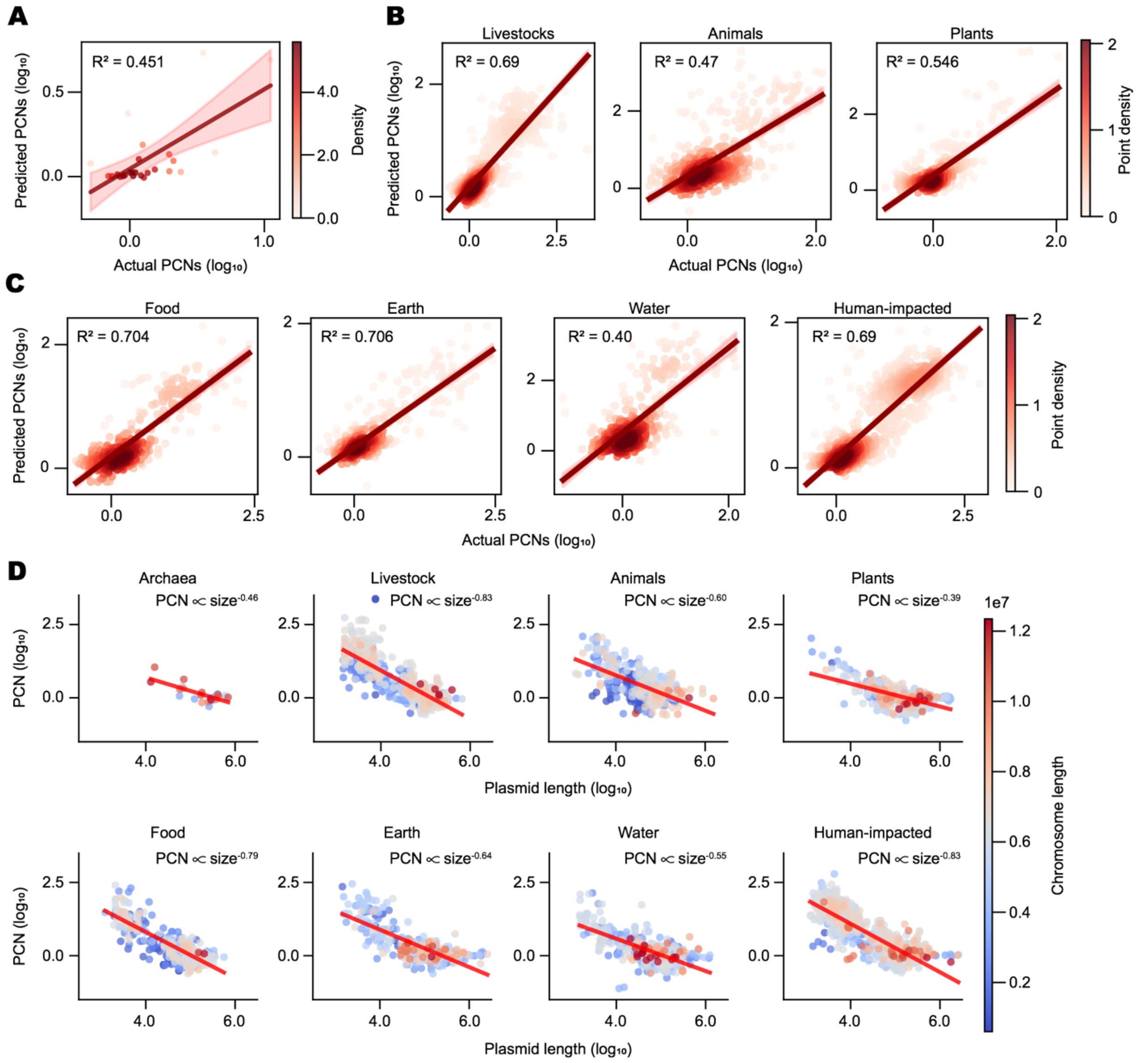

Figure S8. Cross-domain generalization of the PCN prediction model and environment-specific scaling relationships.

(A) Cross-domain predictive performance. Predicted versus true PCNs for archaeal plasmids, using a model trained exclusively on bacterial data ($R^2 = 0.451$). The result demonstrates moderate but non-trivial predictive transferability across biological domains. Point density is indicated by color shading.

(B-C) Cross-environment predictive performance from a model trained on human-associated plasmids. Predictions show high accuracy for related environments (livestock, food, human-

impacted, earth: $R^2 = 0.69–0.83$) but reduced for distant contexts (animals, water, plants: $R^2 = 0.40–0.55$). Point density is indicated by color shading.

(D) Domain- and environment-specific power-law relationship between plasmid size and PCN. All scaling relationships are statistically significant ($p < 0.001$). Archaeal plasmids exhibit a shallower scaling exponent ($k = -0.46$) compared to bacterial plasmids. Among environments, human-associated niches show steeper scaling ($k = -0.83$), indicating a stronger size-dependent constraint, while natural environments display more moderate exponents ($k = -0.34$ to -0.55). Individual plasmids are colored by host chromosome length; red lines represent fitted log-log regressions.

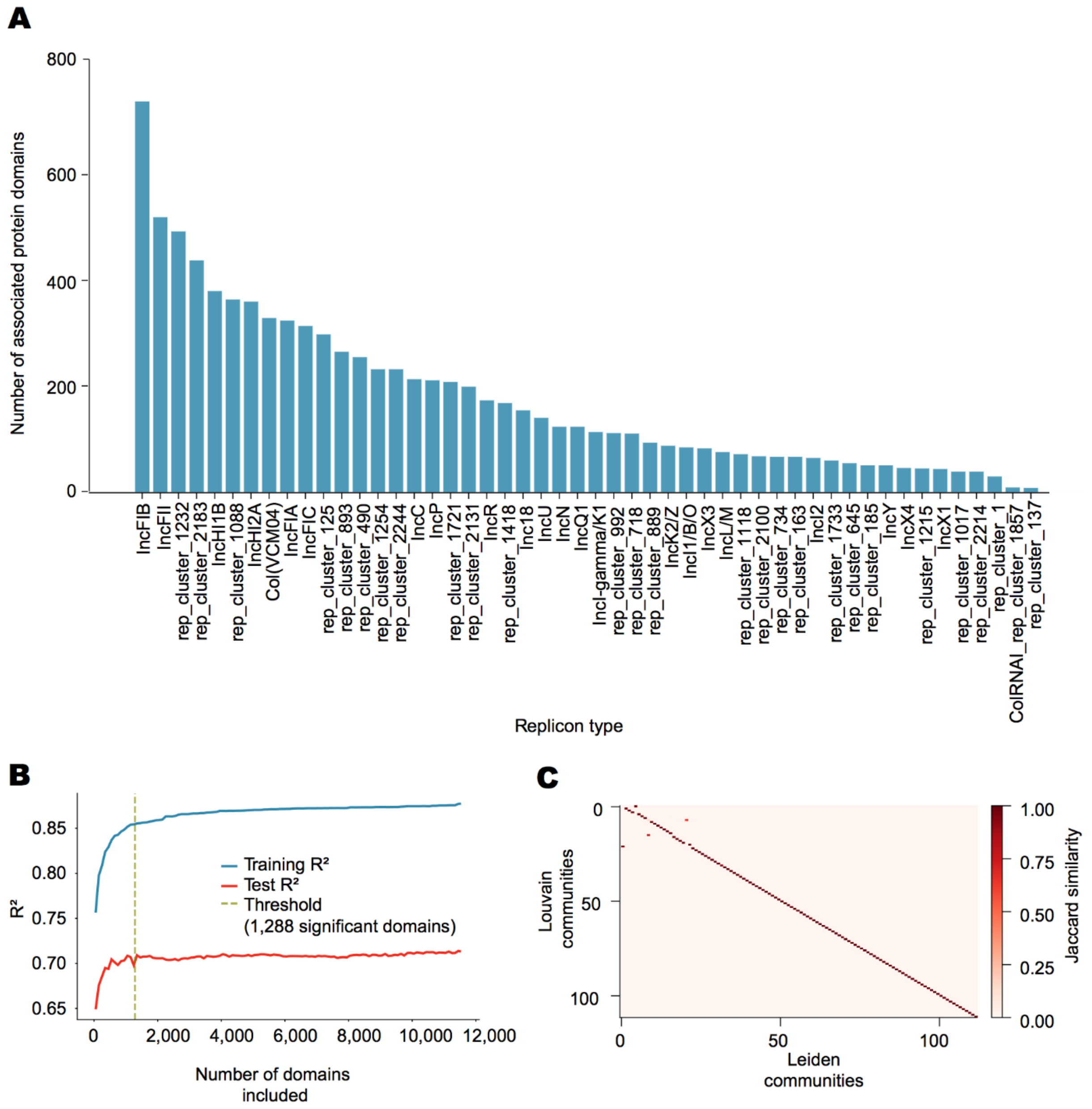

Figure S9| Protein domain associations with replicon types and community detection robustness.

(A) Distribution of significantly associated protein domains across replicon types. Bar heights represent the number of protein domains significantly enriched for each replicon type (FDR-corrected $p < 0.05$), demonstrating that replicon identity strongly structures protein domain composition.

(B) Model performance as a function of the number of domain feature inclusded. Predictive accuracy (test R^2, red line) and model fit (training R^2, blue line) are plotted against the cumulative

number of domains, ranked by significance of their association with PCNs. Performance plateaus after the inclusion of domains with significant PCN associations (n = 1,288, vertical line), validating the feature selection strategy.

(C) Robustness of plasmid community detection to algorithmic method. A heatmap displays the Jaccard similarity between community assignments generated by the Louvain and Leiden algorithms (112 communities each). High average similarity (99.5%) and a large number of perfectly matched communities (n = 89) confirm that the inferred community structure is not an artifact of the specific clustering algorithm used.

Reference

1 Xue, W., Hong, J. & Wang, T. The evolutionary landscape of prokaryotic chromosome/plasmid balance. *Communications Biology* **7**, 1434 (2024).
