# Supplementary material for: Integrating theory and machine learning to reveal determinants of plasmid copy number
Source: Nat Commun. 2026 Apr 22;17:5539. doi: 10.1038/s41467-026-72303-0 (PMC13287686; doi:10.1038/s41467-026-72303-0)
Supplement: Supplementary file 3 — Reporting Summary [file 41467_2026_72303_MOESM3_ESM.pdf]

nature portfolio

Corresponding author(s): Teng Wang

Last updated by author(s): Apr 1, 2026

Reporting Summary

Nature Portfolio wishes to improve the reproducibility of the work that we publish. This form provides structure for consistency and transparency in reporting. For further information on Nature Portfolio policies, see our Editorial Policies and the Editorial Policy Checklist.

Statistics

For all statistical analyses, confirm that the following items are present in the figure legend, table legend, main text, or Methods section.

n/a	Confirmed	
☐	☒	The exact sample size (*n*) for each experimental group/condition, given as a discrete number and unit of measurement
☐	☒	A statement on whether measurements were taken from distinct samples or whether the same sample was measured repeatedly
☐	☒	The statistical test(s) used AND whether they are one- or two-sided<br>*Only common tests should be described solely by name; describe more complex techniques in the Methods section.*
☐	☒	A description of all covariates tested
☐	☒	A description of any assumptions or corrections, such as tests of normality and adjustment for multiple comparisons
☐	☒	A full description of the statistical parameters including central tendency (e.g. means) or other basic estimates (e.g. regression coefficient) AND variation (e.g. standard deviation) or associated estimates of uncertainty (e.g. confidence intervals)
☐	☒	For null hypothesis testing, the test statistic (e.g. *F*, *t*, *r*) with confidence intervals, effect sizes, degrees of freedom and *P* value noted<br>*Give P values as exact values whenever suitable.*
☒	☐	For Bayesian analysis, information on the choice of priors and Markov chain Monte Carlo settings
☒	☐	For hierarchical and complex designs, identification of the appropriate level for tests and full reporting of outcomes
☐	☒	Estimates of effect sizes (e.g. Cohen's *d*, Pearson's *r*), indicating how they were calculated

Our web collection on statistics for biologists contains articles on many of the points above.

Software and code

Policy information about availability of computer code

Data collection	Python 3.11.3, Prodigal V2.6.3, Pfam database v37.1, HMMScan v3.4, scikit-learn V1.5.2, XGBoost V2.1.1, Cytoscape V3.10.3, Comprehensive Antibiotic Resistance Database (CARD v4.0.0), Resistance Gene Identifier (RGI v6.0.3), Mob-typer V3.1.9, see https://github.com/Iqra123isynbio/Plasmid_copy_number_Prediction.
Data analysis	Python 3.11.3, see https://github.com/Iqra123isynbio/Plasmid_copy_number_Prediction.

For manuscripts utilizing custom algorithms or software that are central to the research but not yet described in published literature, software must be made available to editors and reviewers. We strongly encourage code deposition in a community repository (e.g. GitHub). See the Nature Portfolio guidelines for submitting code & software for further information.

Data

Policy information about availability of data

All manuscripts must include a data availability statement. This statement should provide the following information, where applicable:

- Accession codes, unique identifiers, or web links for publicly available datasets
- A description of any restrictions on data availability
- For clinical datasets or third party data, please ensure that the statement adheres to our policy

All the data associated with this work are available at the Github repository (https://github.com/Iqra123isynbio/Plasmid_copy_number_Prediction).

Research involving human participants, their data, or biological material

Policy information about studies with human participants or human data. See also policy information about sex, gender (identity/presentation), and sexual orientation and race, ethnicity and racism.

Reporting on sex and gender	Not applicable.
Reporting on race, ethnicity, or other socially relevant groupings	Not applicable.
Population characteristics	Not applicable.
Recruitment	Not applicable.
Ethics oversight	Not applicable.

Note that full information on the approval of the study protocol must also be provided in the manuscript.

Field-specific reporting

Please select the one below that is the best fit for your research. If you are not sure, read the appropriate sections before making your selection.

[x] Life sciences [ ] Behavioural & social sciences [ ] Ecological, evolutionary & environmental sciences

For a reference copy of the document with all sections, see nature.com/documents/nr-reporting-summary-flat.pdf

Life sciences study design

All studies must disclose on these points even when the disclosure is negative.

Sample size	To build up the machine learning model, we analyzed a dataset comprising 11,338 fully assembled plasmid sequences from 4,317 prokaryotic genomes with computationally determined copy number estimates from Maddamsetti et al. To assess the copy number distribution of clinically relevant plasmids, we obtained genomic data from NCBI's Pathogen Detection resource on May 02, 2025. We identified 30,254 plasmids from 10,664 genome assemblies, with the remaining genomes containing no detectable plasmids. For plasmids in IMG/PR database, our quality control process yielded a final dataset of 154,680 high-quality plasmid sequences from an initial collection of 699,978 entries.
Data exclusions	For clinical plasmids, we applied stringent filters to select only “complete” genomes from “clinical” isolates. For plasmids in IMG/PR database, we excluded sequences containing more than 5% ambiguous bases and removed all plasmids shorter than 1 kb. Additionally, we only selected those plasmids labeled as "putatively complete". We took these filtering steps to promote the reliability of our analysis.
Replication	We evaluated the performance of our machine learning model using ten random splits of the dataset, with training and test sets divided in a 4:1 ratio.
Randomization	No experimental groups in this study.
Blinding	No group allocation in this study.

Reporting for specific materials, systems and methods

We require information from authors about some types of materials, experimental systems and methods used in many studies. Here, indicate whether each material, system or method listed is relevant to your study. If you are not sure if a list item applies to your research, read the appropriate section before selecting a response.

Materials & experimental systems

n/a	Involved in the study	
[x]	[ ]	Antibodies
[x]	[ ]	Eukaryotic cell lines
[x]	[ ]	Palaeontology and archaeology
[x]	[ ]	Animals and other organisms
[x]	[ ]	Clinical data
[x]	[ ]	Dual use research of concern
[x]	[ ]	Plants

Methods

n/a	Involved in the study	
[x]	[ ]	ChIP-seq
[x]	[ ]	Flow cytometry
[x]	[ ]	MRI-based neuroimaging

Plants

Seed stocks	Not applicable.
Novel plant genotypes	Not applicable.
Authentication	Not applicable.
