# Supplementary material for: Integrating theory and machine learning to reveal determinants of plasmid copy number
Source: Nat Commun. 2026 Apr 22;17:5539. doi: 10.1038/s41467-026-72303-0 (PMC13287686; doi:10.1038/s41467-026-72303-0)
Supplement: Supplementary file 2 — Peer Review file [file 41467_2026_72303_MOESM2_ESM.pdf]

nature portfolio

Peer Review File

Integrating theory and machine learning to reveal determinants of plasmid copy number

Corresponding Author: Professor Teng Wang

This file contains all reviewer reports in order by version, followed by all author rebuttals in order by version.

Version 0:

Reviewer comments:

Reviewer #1

(Remarks to the Author)
General Assessment.
The manuscript presents technically sound work that combines theoretical modeling, machine learning, and metagenomic analyses to predict plasmid copy number (PCN). However, the novel contributions are limited, and more critically, the biological conclusions lack the necessary context to provide genuine insight into plasmid ecology.

The work demonstrates several commendable attributes: clear writing, informative figures, a theoretical framework explaining the power-law relationship between plasmid size and PCN, and integration of both molecular (protein domains) and ecological features. The analytical scale is impressive (>700,000 plasmid sequences from IMG/PR, >10,000 trained plasmids).

Limited Novelty.
The inverse power-law relationship between plasmid size and PCN is not original; previous studies have already established this correlation. The theoretical model, while useful, primarily formalizes an already-evident empirical relationship. The application of machine learning with plasmid features represents an incremental rather than transformative advance.

The Fundamental Problem: PCN Divorced from Host Context The core limitation is that the work treats PCN as an intrinsic plasmid property, when in reality it is fundamentally determined by host-plasmid interactions. The manuscript characterizes plasmids by size, protein domains, and ecological environment, but these features provide little additional biological insight beyond plasmid family classification.

Plasmid copy number is overwhelmingly determined by the plasmid family itself—specifically the replication and segregation systems it encodes. The distinction between low-copy families (e.g., pSC101: ~2–5 copies) and high-copy families (e.g., pBR322: ~100–300 copies) reflects fundamental biological differences in replication control, not nuanced variations predictable from secondary molecular features.

Critically, the work neglects that the same plasmid exhibits different PCN depending on its bacterial host. This host-dependence is not merely noise; it reflects the host's physiology, cell cycle regulation, and metabolic capacity—factors that fundamentally determine whether high PCN is maintained or selected against. Without decomposing this host effect, PCN measurements from metagenomic data remain ecologically decontextualized.

The ecological analysis is similarly superficial. The work identifies "hotspots" of elevated PCN in specific niches but does not consider that these niches harbor fundamentally different bacterial populations. The ecological context of PCN cannot be interpreted without knowing which bacterial taxa dominate each environment and how their physiology constrains plasmid replication rates. PCN values become meaningless when divorced from the host populations that maintain them.

Conclusion
In its current form, the work effectively documents patterns but fails to provide true predictive insight into plasmid biology. PCN as an isolated parameter has limited utility without understanding the three inseparable factors that determine it:

Plasmid family (replication/segregation system)
Host identity (physiology and cell cycle)
Selective pressures (ecological and competitive context)

To advance from descriptive analysis to meaningful biological contribution, the manuscript must explicitly contextualize PCN within the dynamics of host-plasmid-environment interactions rather than treating it as an intrinsic plasmid property.

Other minor observation:

Line 75: The citations in this article are duplicated, as they cite both BioRxiv and the published paper on all occasions.
Lines 86-87: "Plasmid length varied significantly, ranging from 1 kb to 2.59 Mb…" It is quite rare to find 2.59 Mb plasmids; in these cases, the boundary between "plasmid" and "secondary chromosome" is very blurred, and this type of plasmid should still be filtered out.
S1-A: The regression points/lines are not visible due to the X-axis scale, which can be better adjusted.
FigS4-E: The X-axis is missing.
Fig 4F: There is a typo in Y axis.
Fig5B and Line 281: It is not understood whether they are synthetic plasmids or plasmids derived from synthetic environments.
Line 349-350: "The same plasmid may exhibit slightly different copy numbers in different hosts, further complicating PCN prediction". More information would be needed to demonstrate that the PCN actually change between different hosts.
Line 401. Why transform data with log1

(Remarks on code availability)

Reviewer #2

(Remarks to the Author)
This manuscript presents an integrated theoretical and machine-learning framework for understanding and predicting plasmid copy number (PCN) across diverse microbial taxa. The authors first derive an analytical model that explains the empirically observed inverse power-law relationship between plasmid size and copy number, based on opposing host-level and plasmid-level selective pressures. They then develop a random-forest regression model incorporating plasmid sequence composition (k-mers), size, host genome length, and, importantly, plasmid-encoded Pfam domains, achieving substantially improved predictive accuracy. Application to large-scale clinical and environmental datasets reveals taxonomic, geographic, and ecological patterns in PCN and antibiotic-resistance gene (ARG) dosage.

I found the paper conceptually strong, logically structured, and methodologically rigorous. It is an interesting study that combines quantitative theory and machine learning to bridge an important gap in microbial genomics. I genuinely enjoyed reading it.

Comments

Interpretation of the power-law scaling
From my background in physics, I find the claim of power-law scaling particularly intriguing but also somewhat ambiguous. The authors report an experimental exponent of –0.71, but the underlying data appear noisy, and it is not entirely clear to me that they really follow a power-law. It would help if the authors could provide a clearer statistical justification for the claimed scaling. (minor note: it would be helpful to bin the data (Fig. 1a) and plot the mean in each bin).

Furthermore, the text mentions that the theoretical model predicts an exponent of –1, and that the difference is attributed to neglected biological factors (e.g., gene heterogeneity, plasmid–chromosome conflicts). However, it remains unclear how and why these specific factors quantitatively alter the scaling exponent (it will probably affect the pre-factor). This raises a broader conceptual question: is the scaling universal? I tend to believe it is not, which could justify the authors' subsequent focus on incorporating more biological detail through a machine-learning approach.

Correlational versus mechanistic claims
The section on gut-specific adaptation of high-copy plasmids is fascinating but currently reads as a mechanistic conclusion. Given that these inferences are based solely on correlations in predicted PCN and domain enrichments, it would be more appropriate to frame them as hypotheses or candidate mechanisms rather than causal explanations.

Dataset bias and generalization
The training dataset is dominated by Enterobacteriaceae (43%) and human-associated plasmids (~38%), with archaeal and environmental plasmids underrepresented. The authors acknowledge this, but it remains unclear how archaeal or environmental plasmids might exhibit different behaviors. A short discussion on this point would help delineate the model's applicability and limitations.

(Remarks on code availability)

Reviewer #3

(Remarks to the Author)
The manuscript "Integrating theory and machine learning to reveal determinants of plasmid copy number" describes a theoretical derivation of plasmid copy number (PCN) from first principles and a machine learning approach utilizing plasmid features for prediction of the plasmid copy number. The presentation of a simple theory representing established relationships between plasmid size and copy number is presented as a plausible but ineffective method for prediction of PCN, which is then enhanced using the proposed machine learning model. The underlying methodology offers a novel approach in prediction of plasmid copy number in plasmids from metagenomic datasets (where read depth comparison to the host chromosome cannot necessarily be performed) as well as assessment of model features which contribute to prediction of PCN. The application of the model to datasets of clinical and metagenomic plasmids offered application and biological interpretation of the model in order to highlight its usefulness.

This manuscript is well written and provides a succinct description of the results and methods used. Methodology for dataset selection and machine learning approaches are sound and supported by relevant references. Associated code for all aspects of the study is both provided and documented on Github effectively.

This manuscript provides a well-described methodology for PCN prediction and analysis of potential uses and application of the method to important issues such as horizontal gene transfer of antimicrobial resistance genes. However, several concerns regarding the manuscript are listed below.

Comments and concerns:
1. In relation to the testing of feature selection impact on model accuracy, the model utilizing domains, plasmid length, and chromosomal length was described as having the highest accuracy. However, the two models presented for downstream application of PCN estimation both included k-mers as elements of the feature set. If these k-mer features were not included in the model which had maximal accuracy in testing, what is the reasoning for including them in them in two models (or at least the one which intends to use chromosomal data)?

2. Why were k-mers selected of lengths 1,2 and 3? Were longer k-mers considered in order to include additional sequence context and information (including key non-coding plasmid regions such as the origin of replication) at the cost of computational complexity? Would a simpler measure of sequence composition (such as G/C%) provide similar or reduced performance in the model?

3. Three replicates were used for testing of feature combinations in the model. The error presented in Figure S2 and Figure 2B present notable deviation in performance between these replicate runs. Additional replicate runs in order to better define the error of performance across feature combinations would improve resolution of potential differences (or lack thereof) among the similar model performance for different feature combinations.

4. The Louvain algorithm was selected for community detection based on the protein domain content of plasmids. The Leiden algorithm was developed for community detection and has been shown to offer improvements in community detection and assurance of connectedness among detected communities as compared to the Louvain algorithm.

Citation for Leiden algorithm:
Traag, V.A., Waltman, L. & van Eck, N.J. From Louvain to Leiden: guaranteeing well-connected communities. Sci Rep 9, 5233 (2019). https://doi.org/10.1038/s41598-019-41695-z

5. The provided codebase contains code relating to recreation of the model and provides the model as files to be loaded for downstream use. However, the example which is provided in the repository for user testing of the model is 4A/Clinical_isolates-PCN_predictions.ipynb. This file relies on Figure_4_data.csv in order to test the model's performance on data from the clinical isolates in the study. This file does not appear to contain the columns relating to the necessary feature datapoints to run this example file (does not include the k-mers, domain presence and absence, etc.). Inclusion of an example data file for end users (which is what the github documentation implies) would be beneficial for users interested in using the provided model and code. Additionally, an environment file for installation of necessary packages or enumeration of necessary installations in the repository's readme would improve the usefulness of the provided code.

Minor comments:
1. Line 30: Please define the term ARG (presumably antibiotic resistance genes) at its first use.

2. Line 25: Listing the specific number of plasmids used in model training may be preferable to ">10,000".

3. Line 388-394: Does inclusion of additional domains (through relaxation of statistical filtration) affect model performance?

4. Line 408-411: Several alternatives to the selected random forest model were stated, however no performance statistics or citations reinforcing the optimality of this selection were offered.

5. Inclusion of additional details of the pre-evaluation process would improve methodological clarity.

6. Line 411-414: Optimal model hyperparameters are clearly calculated in the provided code and the process is

documented. Were these optimal parameters for the provided models stated in the manuscript or supplementary material?

7. Line 464: There is an extra space in “1, 288”.

8. Line 521: There appears to be a missing citation for Prokka.
Citation: Torsten Seemann, Prokka: rapid prokaryotic genome annotation, Bioinformatics, Volume 30, Issue 14, July 2014, Pages 2068–2069, https://doi.org/10.1093/bioinformatics/btu153

9. Some references do not contain proper usage of italics for genus and species names (such as Yersinia in reference 7).

10. This does not necessarily merit a change, but the order of panels in Figure 1, specifically with Figure 1G being placed in the upper right corner, is somewhat visually confusing.

11. Figure 3D: Addition of the size of each community to the figure would improve interpretation.

12. Similar to Figure 1, as this does not necessarily merit change, but the placement of panel I above panel H is somewhat visually confusing.

(Remarks on code availability)
The code provided includes both the calculated machine learning model and the code necessary to generate the model and analysis necessary for generation of manuscript figures. A README is included detailing the contents of the repository. Several concerns regarding the inclusion of example data and package installation instructions are listed in the comments to the author, however the provided code appears to be sufficient for recreation of the presented analysis and the provided model is available for use for the community.

Version 1:

Reviewer comments:

Reviewer #1

(Remarks to the Author)
Point-to-point response to authors:

I thank the authors for their detailed responses and for the substantial effort in revising the manuscript. The addition of the sensitivity analysis for large plasmids and the CQV calculations certainly strengthen the technical rigor of the work. I also appreciate the authors’ candid acknowledgment that the primary aim of this study is methodological: to establish a predictive baseline for plasmid copy number (PCN) in the absence of host information.

However, while the technical execution remains commendable, the biological and ecological contextualization is still insufficient. The core conceptual limitations have not been fully resolved, and the manuscript continues to conflate statistical predictive accuracy with genuine biological insight. For the manuscript to be suitable for publication, the following discrepancies must be addressed:

The Fundamental Problem - Host Taxonomy is Not Host Physiology.
The authors argue that host-dependent variation is not the primary source of PCN variance, citing that the inclusion of host genus yielded a modest predictive improvement (
Δ
R
2
≈
0.006
ΔR
2
≈0.006) and that 88.7% of replicon types showed no significant PCN variation across genera. However, taxonomic classification at the genus level is fundamentally inadequate to capture host physiology. In situ PCN is dynamically shaped by strain-level variations, metabolic states, and environmental stress—factors that are completely invisible at the broad genus level. Dismissing the host's effect based on genus-level averages does not resolve the reality that PCN is an emergent property of host-plasmid-environment interactions. The manuscript must explicitly acknowledge that the apparent "host-independence" in this model is largely an artifact of coarse taxonomic resolution, rather than a proven biological reality.

Predictive Accuracy vs. Biological Relevance of Replicon Families.
The authors successfully demonstrate that their integrated domain-centric model statistically outperforms a replicon-only baseline, capturing intra-family variation (via the CQV analysis). However, from an ecological standpoint, the manuscript fails to demonstrate that predicting an exact numerical PCN provides a substantial biological advantage over simply identifying the plasmid family. Plasmid replicon families inherently dictate the overarching replication strategy (i.e., broad low- vs. high-copy categories), which is largely sufficient to draw the broad ecological patterns presented in this study. The incremental precision provided by the machine-learning model over this categorical baseline does not translate into

transformative ecological insight. Furthermore, without host physiological data, it is impossible to definitively conclude whether this precise intra-family variance is truly driven by secondary molecular features or is merely a confounding artifact of host strain and physiological differences within the dataset. The manuscript must critically address the limited biological utility of this numerical refinement compared to replicon classification.

Superficial Ecological Claims in the Abstract.
I appreciate that the authors have appropriately re-framed the ecological analysis in the revised Discussion (lines 427-434), accurately defining these findings as "hypothesis-generating" and acknowledging that current limitations in host taxonomic resolution constrain definitive ecological interpretation. However, this cautious and necessary framing is entirely absent from the Abstract. The Abstract continues to claim that the study uncovers "niche specific taxonomic PCN hotspots and ecological adaptations" (lines 28-30) and provides "critical insights into plasmid ecology." To advance to publication, these strong ecological claims in the Abstract and Introduction must be explicitly toned down to align with the methodological nature of the study and the more realistic, constrained scope established in the revised Discussion.

In conclusion, the analytical scale of this study remains impressive, but to provide a meaningful biological contribution, the manuscript must ensure that its introductory and concluding claims are strictly constrained by its methodological limitations.

(Remarks on code availability)

Reviewer #2

(Remarks to the Author)
I am happy with the revision.

(Remarks on code availability)

Reviewer #3

(Remarks to the Author)
The manuscript “Integrating theory and machine learning to reveal determinants of plasmid copy number” and the adjustments made to the original submission of the manuscript have resulted in significant improvements to the quality of the work. Previous concerns raised regarding the manuscript were widely addressed, including: clarification of the methodology regarding feature selection, improvements to robustness of testing framework for feature selection and model evaluation (number of replicates for feature selection, etc.), and availability of data for execution of code provided with the manuscript. Improvements to the methodology described for the machine learning framework bolster the results described in the paper. Improvements to wording based on reviewer comments have also improved the clarity of the results. Overall, this manuscript is of high quality and presents meaningful research in the field of plasmid copy number prediction and evaluation which should be of use to the community. Several minor comments are listed below.

Minor Comments:
Line 546: Citation number (58 in the current manuscript) for use of scikit-learn’s implementation of cosine similarity is missing.

Lines 571-575: Presumably default values for confidence thresholds and alignment coverage were used for MOB-typer. It would be useful to state this in the methods section.

(Remarks on code availability)
The code remains in a state which is well documented and clearly reflects the analysis described in the manuscript. Additional clarification of installation and protocol for use of code by end users and availability of the full dataset necessary for use of the code are now provided, as well. This appropriately addresses the previous concerns raised regarding the availability of code and data for the manuscript.

Open Access This Peer Review File is licensed under a Creative Commons Attribution 4.0 International License, which permits use, sharing, adaptation, distribution and reproduction in any medium or format, as long as you give appropriate credit to the original author(s) and the source, provide a link to the Creative Commons license, and indicate if changes were made.
In cases where reviewers are anonymous, credit should be given to 'Anonymous Referee' and the source.
The images or other third party material in this Peer Review File are included in the article's Creative Commons license, unless indicated otherwise in a credit line to the material. If material is not included in the article's Creative Commons license and your intended use is not permitted by statutory regulation or exceeds the permitted use, you will need to obtain permission directly from the copyright holder.
To view a copy of this license, visit https://creativecommons.org/licenses/by/4.0/

Point-to-point response to reviewer's comments:

Reviewer #1: The manuscript presents technically sound work that combines theoretical modeling, machine learning, and metagenomic analyses to predict plasmid copy number (PCN). However, the novel contributions are limited, and more critically, the biological conclusions lack the necessary context to provide genuine insight into plasmid ecology. The work demonstrates several commendable attributes: clear writing, informative figures, a theoretical framework explaining the power-law relationship between plasmid size and PCN, and integration of both molecular (protein domains) and ecological features. The analytical scale is impressive (>700,000 plasmid sequences from IMG/PR, >10,000 trained plasmids).

We sincerely thank the reviewer for the careful and constructive evaluation of our manuscript, and for recognizing its technical rigor, analytical scale, and clarity of presentation. We particularly appreciate the reviewer's thoughtful comments regarding the need to better articulate the conceptual novelty and to place our biological findings in a richer ecological context. In response to these important concerns, we have substantially revised the manuscript by performing a number of new analyses, adding more discussions and integrating recent literature. We believe these changes significantly strengthen the biological insights and contextual relevance of the study. All points raised by the reviewer have been carefully addressed in the revised manuscript, as detailed below.

Limited Novelty: The inverse power-law relationship between plasmid size and PCN is not original; previous studies have already established this correlation. The theoretical model, while useful, primarily formalizes an already-evident empirical relationship. The application of machine learning with plasmid features represents an incremental rather than transformative advance.

We thank the reviewer for this important comment and agree that the inverse power-law relationship between plasmid size and copy number has been reported in recent studies[1,2], **as we have clearly acknowledged in the original manuscript.** Our goal was not to re-establish this empirical correlation, but to address key conceptual and practical gaps that those studies explicitly identified.

First, while prior works observed the size–PCN power law, its origin remained unclear. For instance, in a recent paper, Ramiro-Martínez *et al.* explicitly pointed out 'Arguably, the most intriguing result of our work is that a PCN-size scaling law governs plasmid biology across bacterial species…**However, the underlying molecular mechanism remains to be uncovered**'[2]. In another recent paper, Maddamsetti *et al.* also observed the PCN-size scaling and pointed out ' **further theory and quantitative experiments**

are needed to understand the biophysical and evolutionary origins of these empirical scaling laws'[1]. Our theoretical framework, which permits an analytical solution, directly addresses this open question by showing that inverse scaling can emerge naturally from a trade-off between host-level selection to minimize metabolic burden and plasmid-level selection to maintain replication competitiveness. Importantly, the model also predicts the additional scaling relationship between chromosome size and plasmid content, which was previously observed empirically but lacked mechanistic interpretation.

Second, the scaling relationship between PCN and plasmid size serves merely as a starting point of our study. PCN prediction based on this relationship alone exhibits limited accuracy. In fact, the scaling relationship only appears conserved at a coarse level but systematically modulated by plasmid-specific biological features. This conceptual gap directly motivates our subsequent machine-learning framework, which incorporates molecular and regulatory details to capture deviations from the mean-field prediction.

Third, although the machine-learning methodology itself builds on established techniques, its biological impact is not incremental. Our model achieves the highest predictive accuracy for plasmid copy number to date, and this predictive power is essential for downstream applications. Accurate PCN estimation is a prerequisite for deciphering ecological patterns of plasmid abundance, environmental adaptation, and antibiotic resistance gene dosage. By enabling reliable PCN inference for hundreds of thousands of metagenomic plasmids—including those lacking host information—our approach makes it possible, for the first time, to map PCN distributions at ecological and clinical scales that are inaccessible to experimental methods.

In this sense, our work complements recent foundational studies: where prior work established robust empirical patterns, we provide a mechanistic explanation, establish a machine-learning framework with enhanced predictive power, and perform metagenomic-scale prediction with direct ecological and clinical relevance. We have revised the Introduction lines 61-75 and Discussion lines 344-352 to clarify this positioning and to better articulate the biological significance of these contributions.

The Fundamental Problem - PCN Divorced from Host Context. The core limitation is that the work treats PCN as an intrinsic plasmid property, when in reality it is fundamentally determined by host-plasmid interactions. The manuscript characterizes plasmids by size, protein domains, and ecological environment, but these features provide little additional biological insight beyond plasmid family classification.

We thank the reviewer for this important comment. We agree that plasmid copy number is, in principle, a property emerging from host–plasmid interactions. Our goal was not to treat PCN as an intrinsic plasmid property, but rather to assess to what extent it can be predicted from diverse features, in particular, plasmid features in the common and methodologically challenging scenarios where host information is absent, such as in metagenomic datasets. We appreciate the opportunity to clarify our approach and to present new analyses that directly address the role of host context.

In light of the reviewer's comment, we performed new analyses by explicitly incorporating host genus as an additional feature in our predictive model. This inclusion led to a statistically significant but modest improvement in performance ($\Delta R^2 \approx 0.006$), indicating that while host identity contributes additional information, the majority of explainable PCN variance is already captured by plasmid-encoded sequence and molecular features. These results are now detailed in the revised Methods (lines 510–517, Supplementary Fig. S2D).

This result is consistent with recent large-scale comparative analyses (Ramiro-Martínez et al., *Nature Communications* 2025)[2], which show that while different hosts harbor different plasmid repertoires, the PCN of individual plasmids is largely stable across hosts, with notable host-dependent effects confined to a small minority of replicon types and of limited magnitude. **We independently validated this pattern in our dataset: most replicon types exhibited no significant PCN variation across different host genera, and among those that did, only 5.1% of host transitions resulted in a detectable PCN shift** (Methods, lines 571–586; Supplementary Fig. S7A).

While plasmid family (defined by replicon type) is an important determinant of PCN[2], our analysis demonstrates that molecular features provide additional predictive power. To evaluate the contribution of our framework beyond broad family classification, we trained another model that predicts PCN based solely on replicon type. When directly compared, the integrated domain-centric model significantly outperformed this replicon-only baseline. This performance gain confirms that the detailed molecular architecture captured by our model—including protein domains, sequence composition, and size—captures the fine-scale regulatory variation that determines precise PCN, moving beyond the categorical distinctions provided by replicon family alone (Supplementary Fig. S2F, lines 599-607).

We have also expanded the Discussion (lines 385–407) to explicitly address the contribution of host context and to frame PCN determination as a composite of plasmid-intrinsic mechanisms and host-dependent modulation. We believe these clarifications and additional analyses directly engage with the reviewer's concern and reinforce the validity and applicability of our approach.

Plasmid copy number is overwhelmingly determined by the plasmid family itself—specifically the replication and segregation systems it encodes. The distinction between low-copy families (e.g., pSC101: ~2–5 copies) and high-copy families (e.g., pBR322: ~100–300 copies) reflects fundamental biological differences in replication control, not nuanced variations predictable from secondary molecular features.

We appreciate the reviewer's comments. We agree with the reviewer that plasmid family (replicon type) provides a critical baseline for PCN, reflecting fundamental differences in replication and segregation strategies between low- and high-copy plasmids. Our work does not dispute this point.

Our objective, however, is to move beyond this categorical distinction to predict the quantitative PCN value. As recent large-scale studies noted, replicon family alone does not fully determine quantitative PCN (Ramiro-Martínez et al., *Nature Communications* 2025)[2]. In particular, substantial copy-number variation exists within several well-defined families—most notably among high-copy Col-like plasmids. This indicates that while family provides the essential strategy, the precise copy number is modulated by finer-scale genetic determinants.

In light of the reviewer's comment, we also performed additional analysis to validate this within-family variation using our dataset. Analyzing replicon families with ≥30 plasmids, we calculated the Coefficient of Quartile Variation (CQV) for each family. Among high-variation families (CQV > 0.5), we observed: IncQ1 (CQV=0.763, n=90), ColpVC (CQV=0.691, n=59), and IncP (CQV=0.655, n=85) (lines 587-591, Supplementary Fig. S7B). These results confirm Ramiro-Martínez et al.'s observation that replicon family classification alone cannot fully explain quantitative PCN variation.

We emphasize that protein domains and replication control are mechanistically linked, not conflicting. Replication systems are implemented by proteins whose functions are defined by conserved domains. To test this explicitly, we performed domain–replicon enrichment analysis across 1,288 protein domains and 64 prevalent replicon types. We identified 9,027 significant associations (FDR-corrected), with 98.9% of domains (1,274/1,288) significantly linked to at least one replicon type (lines 592-598, Supplementary Fig. S9A). This confirms that protein domain features implicitly encode replication control information.

Therefore, our model uses domain features to quantify the subtle regulatory variations *within* a given replication strategy that fine-tune PCN. This approach offers two key advantages: 1) it resolves PCN differences among plasmids of the same family, and 2) it enables predictions for plasmids where family assignment is ambiguous (e.g., multi-replicon plasmids or novel plasmids).We have incorporated these analyses and

clarifications to underscore that our domain-based framework complements and refines the essential biological insight provided by plasmid family classification.

Critically, the work neglects the same plasmid exhibits different PCN depending on its bacterial host. This host-dependence is not merely noise; it reflects the host's physiology, cell cycle regulation, and metabolic capacity—factors that fundamentally determine whether high PCN is maintained or selected against. Without decomposing this host effect, PCN measurements from metagenomic data remain ecologically decontextualized.

We thank the reviewer for this important comment on the role of host context in determining PCN. We fully agree that host physiology, cell cycle regulation, and metabolic capacity can, in principle, influence PCN and that these interactions are a vital part of plasmid ecology. We did not intend to dismiss these effects; rather, our work aimed to address a common practical challenge: in the vast majority of datasets, detailed information about the host's physiological state is unavailable.

Our methodological focus, therefore, was to assess the predictive power of plasmid-encoded features in the absence of host data—a scenario that reflects the reality of metagenomic studies. Our analyses were designed to quantify how much of the observable PCN variation can be attributed to the plasmid itself.

The empirical results from our study, corroborated by recent large-scale work, suggest that host-dependent variation, while biologically meaningful in many instances, is not the primary source of PCN variance across the plasmid landscape. As detailed in our previous response, the inclusion of host genus in our model yielded a statistically significant but very modest improvement in predictive power ($\Delta R^2 \approx 0.006$). Furthermore, our systematic analysis across 6,286 plasmids found that most replicon types (88.7%) exhibited no statistically significant PCN variation across different host genera.

We believe these findings offer a valuable ecological perspective. By establishing a robust baseline prediction for PCN, our model provides an ecologically meaningful null expectation. Significant deviations from this baseline in specific host-plasmid combinations—which we acknowledge as biologically meaningful signals rather than mere noise—become interpretable as indicators of unique adaptation, stress, or interaction, consistent with the host-dependent effects emphasized by the reviewer. This approach shifts the analysis from a decontextualized PCN value to an interpretative framework where measured PCN is assessed relative to its plasmid-determined expectation, effectively contextualizing it within a potential host environment.

We have clarified this framing in the revised Discussion (lines 385–407), emphasizing that our model provides a baseline for identifying and investigating the host-dependent interactions that are important to a complete understanding of plasmid ecology.

The ecological analysis is similarly superficial. The work identifies "hotspots" of elevated PCN in specific niches but does not consider that these niches harbor fundamentally different bacterial populations. The ecological context of PCN cannot be interpreted without knowing which bacterial taxa dominate each environment and how their physiology constrains plasmid replication rates. PCN values become meaningless when divorced from the host populations that maintain them.

We thank the reviewer for this thoughtful comment and fully agree that plasmid copy number (PCN) is ultimately shaped by the physiological constraints and taxonomic composition of the host populations that maintain plasmids. We appreciate the opportunity to clarify both the scope of our ecological analysis and the intent with which the results should be interpreted.

First, we would like to emphasize that host context was not overlooked in our original analysis. In the original manuscript, we explicitly noted that different environments harbor distinct bacterial communities and that such differences are likely to contribute to the observed variation in PCN distributions (lines 253–254, Supplementary Fig. S4F and G). In response to the reviewer's comment, we have expanded this section by incorporating an analysis of taxonomic composition across clinical niches, stratified by body site and geographic region. This additional analysis reveals clear taxonomic trends, such as the enrichment of *Neisseria gonorrhoeae* in genital samples, which plausibly contributes to the elevated PCN observed in this niche. *N. gonorrhoeae* is also enriched in samples from Russia relative to other regions, helping to explain the high PCN observed there (lines 254-259, Supplementary Fig. S4C and D). These results strengthen the link between ecological patterns and host population structure without altering the central focus of the study.

Second, we agree that a comprehensive, host-resolved ecological interpretation—explicitly modeling which bacterial taxa dominate each environment and how their physiology constrains plasmid replication—represents an important and valuable direction for future research. However, such an analysis lies beyond the primary scope of the present work. The central aim of this study is methodological: to establish and validate a plasmid-centric framework for predicting PCN from sequence-derived features, and to assess whether this framework can reveal coherent, large-scale patterns across heterogeneous ecological contexts. We now clarify this point more explicitly in the revised Discussion and outline host-resolved integration as a natural and necessary next step (lines 424–434).

Finally, we respectfully suggest that PCN patterns can retain ecological meaning even in the absence of fully resolved host population structure:

(1) As noted in our previous response, recent large-scale studies suggest that key plasmid-intrinsic constraints generate robust patterns that persist across diverse hosts. Therefore, our identified "hotspots" reflect environments where plasmids with specific, high-copy genetic architectures are more prevalent—a meaningful finding that generates testable hypotheses.

(2) The systematic differences we observe in PCN distributions, plasmid domain composition and ARG gene dosage across environments reflect real, niche-specific signatures in the plasmid pool. These signatures likely arise from the interplay of plasmid-intrinsic replication strategies and broad environmental selection pressures—a meaningful ecological signal in its own right.

In the revised Discussion, we therefore frame our ecological analysis explicitly as a first-layer, plasmid-centric map: one that delineates broad patterns and generates hypotheses, rather than offering a complete mechanistic explanation (lines 424-434). We believe this positioning appropriately reflects both the strengths and the limitations of the current study, while clearly motivating future work that integrates plasmid-centric models with host-resolved ecological and physiological data.

Conclusion: In its current form, the work effectively documents patterns but fails to provide true predictive insight into plasmid biology. PCN as an isolated parameter has limited utility without understanding the three inseparable factors that determine it: Plasmid family (replication/segregation system); Host identity (physiology and cell cycle); Selective pressures (ecological and competitive context). To advance from descriptive analysis to meaningful biological contribution, the manuscript must explicitly contextualize PCN within the dynamics of host-plasmid-environment interactions rather than treating it as an intrinsic plasmid property.

We thank the reviewer for the comment. The issues raised here—concerning the roles of plasmid family, host context, and ecological selection in shaping PCN—are addressed in detail in our responses to the related comments above and have been incorporated throughout the revised manuscript. Rather than reiterating those analyses here, we refer the reviewer to the corresponding responses and revisions, which collectively reframe PCN as an emergent property of host–plasmid–environment interactions rather than an isolated plasmid attribute.

Other minor observation:

Line 75: The citations in this article are duplicated, as they cite both BioRxiv and the published paper on all occasions.

We thank the reviewer for pointing this out. We have corrected the reference list by removing the duplicated bioRxiv citation and retaining only the corresponding published versions throughout the manuscript.

Lines 86-87: “Plasmid length varied significantly, ranging from 1 kb to 2.59 Mb…” It is quite rare to find 2.59 Mb plasmids; in these cases, the boundary between "plasmid" and "secondary chromosome" is very blurred, and this type of plasmid should still be filtered out.

We thank the reviewer for raising this important point regarding the boundary between large plasmids and secondary chromosomes. To directly address this concern and evaluate its impact on our findings, we performed a sensitivity analysis. We retrained our core prediction model before and after excluding all plasmids larger than 500 kb (n = 189; 1.7% of the original dataset, including the three >2 Mb elements). The results demonstrate that our model’s performance is highly robust to the inclusion or exclusion of these large elements: the predictive accuracy remains highly stable ($R^2 = 0.736 \pm 0.014$) compared to the full dataset model ($R^2 = 0.746 \pm 0.017$; $\Delta R^2 = 0.010$). Furthermore, the PCN predictions for the overlapping plasmid set between the two models were strongly correlated (Pearson’s $r = 0.99$), and all major biological conclusions were unchanged.

We have incorporated this sensitivity analysis as Supplementary Fig. S2G-H and clarified in the lines 533-540 that our results are robust to the inclusion or exclusion of very large plasmids.

S1-A: The regression points/lines are not visible due to the X-axis scale, which can be better adjusted.

We thank the reviewer for the suggestion. We have adjusted the scale in the revised figure, and the regression points/lines are now clearly displayed.

FigS4-E: The X-axis is missing.

We thank the reviewer for pointing it out. In this figure (now Fig. S4G in the updated manuscript), the X-axis corresponds to the sample size, specifically the number of plasmids in each group. We have now revised the figure to include the X-axis label.

Fig 4F: There is a typo in Y axis.

We thank the reviewer for pointing it out. The typo in the Y-axis label of Figure 4F has been corrected in the revised version of the manuscript.

Fig5B and Line 281: It is not understood whether they are synthetic plasmids or plasmids derived from synthetic environments.

We thank the reviewer for highlighting this ambiguity. The "Engineered" category refers to plasmids isolated from engineered or artificial environments (bioreactors, wastewater treatment plants, artificial ecosystems) rather than synthetic laboratory-constructed plasmids. We have clarified this terminology in the revised manuscript (Line 301, Results; Methods section lines 678-680).

Line 349-350: "The same plasmid may exhibit slightly different copy numbers in different hosts, further complicating PCN prediction". More information would be needed to demonstrate that the PCN actually change between different hosts.

We thank the reviewer for this comment. We have provided two examples from previous studies to demonstrate that PCN actually change between different hosts (lines 385-388).

Line 401 - Why transform data with log1

We thank the reviewer for the question. The log(1 + PCN) transformation was applied to address the intrinsic statistical properties of plasmid copy number data. PCN values in our dataset span several orders of magnitude, from a few to several hundred, resulting in a strongly right-skewed distribution. The transformation compresses the dynamic range, making the variance more homogeneous across the scale of PCN values. In addition, the transformation linearizes relationships that are inherently multiplicative, such as power-law relationship. The addition of "+1" is a standard continuity correction that prevents taking the logarithm of zero, which would be mathematically undefined, while having a negligible impact on the transformed values for all PCN > 1. This approach aligns with best practices for modeling count-based genomic data and has been shown to effectively preserve biological signal while improving model fit[3].

We have expanded the explanation in the Methods section (Lines 472-482) to include this detailed justification.

Reviewer #2 (Remarks to the Author): This manuscript presents an integrated theoretical and machine-learning framework for understanding and predicting plasmid copy number (PCN) across diverse microbial taxa. The authors first derive an analytical model that explains the empirically observed inverse power-law relationship between plasmid size and copy number, based on opposing host-level and plasmid-level selective pressures. They then develop a random forest regression model incorporating plasmid sequence composition (k-mers), size, host genome length, and, importantly, plasmid-encoded Pfam domains, achieving substantially improved predictive accuracy. Application to large-scale clinical and environmental datasets reveals taxonomic, geographic, and ecological patterns in PCN and antibiotic resistance gene (ARG) dosage. I found the paper conceptually strong, logically structured, and methodologically rigorous. It is an interesting study that combines quantitative theory and machine learning to bridge an important gap in microbial genomics. I genuinely enjoyed reading it.

We thank the reviewer for the thoughtful and positive evaluation of the manuscript. We are pleased that the reviewer found the study conceptually strong and methodologically rigorous. All constructive suggestions have been carefully addressed in the revised manuscript, as detailed below.

Comments

Interpretation of power-law scaling: From my background in physics, I find the claim of power-law scaling particularly intriguing but also somewhat ambiguous. The authors report an experimental exponent of –0.71, but the underlying data appear noisy, and it is not entirely clear to me that they really follow a power-law. It would help if the authors could provide clearer statistical justification for the claimed scaling. (minor note: it would be helpful to bin the data (Fig1a) and plot the mean in each bin).

We thank the reviewer for this thoughtful and well-taken comment. We agree that claims of power-law scaling require careful statistical justification, particularly given the inherent noise in the raw data.

In response, we have significantly expanded our statistical validation in the revised manuscript (lines 97–103). We conducted three key analyses:

(1) Binned visualization: Following the reviewer's specific suggestion, we have updated Figure 1A to display the mean PCN within logarithmically spaced size bins. This representation effectively reduces visual noise and more clearly reveals the underlying scaling trend.

(2) Bootstrap confidence analysis: To assess the stability and uncertainty of the estimated exponent, we performed bootstrap resampling (1,000 iterations). This analysis yielded a narrow 95% confidence interval for the scaling exponent of (–0.719 to –0.696) (Supplementary Fig. S1A), demonstrating that the –0.71 estimate is robust to sampling variation.

(3) Formal model comparison: To statistically justify the choice of a power-law model over other plausible functional forms, we conducted a formal model comparison using the Akaike Information Criterion (AIC). The power-law model was overwhelmingly favored, with a ΔAIC > 9,000 relative to the alternative models (linear, exponential, and log-linear) (Supplementary Fig. S1B), suggesting that a power-law relationship provides a statistically supported description of the data across the observed range.

Together, these new analyses improve the statistical rigor of our claim and demonstrated that the observed scaling is best described by a power law.

Furthermore, the text mentions that the theoretical model predicts an exponent of –1, and that the difference is attributed to neglected biological factors (e.g., gene heterogeneity, plasmid–chromosome conflicts). However, it remains unclear how and why these specific factors quantitatively alter the scaling exponent (it will probably affect the pre-factor). This raises a broader conceptual question: is the scaling universal? I tend to believe it is not, which could justify the authors' subsequent focus on incorporating more biological detail through a machine-learning approach.

We thank the reviewer for this insightful comment and suggestion. We agree that the analytical model should be interpreted as a mean-field baseline rather than a claim of strict universality.

In our derivation, the exponent of –1 arises under simplifying assumptions: uniform gene density across plasmid sizes, size-independent regulatory costs, functional equivalence of genes in terms of competitiveness, and a constant average metabolic burden per plasmid copy imposed on the host. Under these conditions, copy number scales inversely with plasmid size because each copy contributes proportionally to host cost. The model is intended to demonstrate the *existence* of inverse scaling, not to fix the exponent precisely.

In reality, several biological factors can modify this scaling behavior. Gene-length heterogeneity alters how functional payload scales with plasmid size; plasmid–chromosome conflicts introduce size-dependent replication penalties; and increasingly complex replication and partitioning systems can relax strict proportionality between size and cost. Together, these effects can renormalize the effective scaling exponent.

We therefore agree with the reviewer that the scaling is not strictly universal. Rather, it appears conserved at a coarse level but systematically modulated by plasmid-specific biological features. This conceptual gap directly motivates our subsequent machine-learning framework, which incorporates molecular and sequence details to capture deviations from the mean-field prediction. We have revised the text lines 131-137 to further clarify this interpretation and explicitly frame the analytical model as a baseline rather than a complete mechanistic description.

Correlational versus mechanistic claims. The section on gut-specific adaptation of high-copy plasmids is fascinating but currently reads as a mechanistic conclusion. Given that these inferences are based solely on correlations in predicted PCN and domain enrichments, it would be more appropriate to frame them as hypotheses or candidate mechanisms rather than causal explanations.

We thank the reviewer for this important suggestion. We have revised the section lines 326-327, 336-337 and 339-340 to clearly frame these findings as "correlative patterns generating testable hypotheses" rather than causal explanations. For instance, we now state that gut plasmids are "predicted" to have higher copy numbers and "show enrichment" for specific domains, which "suggests candidate mechanisms" for gut adaptation. This careful wording invites future experimental validation while remaining consistent with our observational data.

Dataset bias and generalization: The training dataset is dominated by Enterobacteriaceae (43%) and human-associated plasmids (~38%), with archaeal and environmental plasmids underrepresented. The authors acknowledge this, but it remains unclear how archaeal or environmental plasmids might exhibit different behaviors. A short discussion on this point would help delineate the model's applicability and limitations.

We thank the reviewer for raising this critical point. To systematically address this concern, we conducted targeted cross-domain and cross-environment generalization tests, which we have now incorporated into the revised manuscript as Supplementary Fig. S8.

Our analyses reveal a nuanced picture:

(1) Cross-domain performance: A model trained exclusively on bacterial plasmids shows a moderate drop in predictive accuracy when applied to archaeal plasmids (R^2 = 0.451). This confirms that while core predictive principles transfer, domain-specific biological variations exist.

(2) Cross-environment performance: Similarly, a model trained on human-associated plasmids generalizes robustly to related niches like livestock ($R^2 = 0.698$), food (0.704), and human-impacted samples (0.699) but shows reduced accuracy for plasmids from animals (0.475) and water (0.404).

(3) Underlying biological variation: Crucially, our power-law scaling analysis provides a plausible explanation for these performance differences. While the fundamental inverse relationship between plasmid size and copy number is universal (all $p < 0.001$), the scaling exponents vary systematically. For instance, archaeal plasmids exhibit a shallower scaling ($k = -0.46$) than bacterial plasmids ($k = -0.70$). Among environments, human-impacted and livestock settings show a steeper slope ($k = -0.83$) compared to plants ($k = -0.39$) and water ($k = -0.55$).

These findings lead to two key conclusions:

(1) Our model successfully captures a core, cross-cutting biophysical principle—the trade-off between plasmid size and copy number—that is evident across all domains.

(2) The quantitative parameters of this relationship are modulated by domain- and environment-specific biology. The steeper slopes in human-associated niches may reflect distinct selection pressures, while different ecological constraints shape the relationship in environmental settings.

Therefore, the current model's predictive power is strongest within the well-sampled bacterial/human-associated domain and serves as a robust baseline. Its decreased accuracy for underrepresented groups is not a failure but a meaningful reflection of biological divergence. We have revised the Discussion (Lines 373-379) to explicitly frame these results, highlighting that the model's performance boundaries themselves offer valuable biological insights and clearly delineating its current applicability. Ultimately, this analysis underscores the need for more balanced sequencing efforts targeting archaeal and environmental plasmids to build truly universal predictive tools.

Reviewer # 3: The manuscript "Integrating theory and machine learning to reveal determinants of plasmid copy number" describes a theoretical derivation of plasmid copy number (PCN) from first principles and a machine learning approach utilizing plasmid features for prediction of the plasmid copy number. The presentation of a simple theory representing established relationships between plasmid size and copy number is presented as a plausible but ineffective method for prediction of PCN, which is then enhanced using the proposed machine learning model. The underlying methodology offers a novel approach in prediction of plasmid copy number in plasmids from metagenomic datasets (where read depth comparison to the host chromosome cannot necessarily be performed) as well as assessment of model features which contribute to prediction of PCN. The application of the model to datasets of clinical and metagenomic plasmids offered application and biological interpretation of the model in order to highlight its usefulness. This manuscript is well written and provides a succinct description of the results and methods used. Methodology for dataset selection and machine learning approaches are sound and supported by relevant references. Associated code for all aspects of the study is both provided and documented on Github effectively. This manuscript provides a well-described methodology for PCN prediction and analysis of potential uses and application of the method to important issues such as horizontal gene transfer of antimicrobial resistance genes. However, several concerns regarding the manuscript are listed below.

We thank the reviewer for their comprehensive and positive evaluation of our manuscript. We are pleased that the reviewer found the methodology sound, the writing clear, and the provided code well-documented. We have carefully considered and addressed all the comments in the revised manuscript and below.

Comments and concerns:

1. In relation to the testing of feature selection impact on model accuracy, the model utilizing domains, plasmid length, and chromosomal length was described as having the highest accuracy. However, the two models presented for downstream application of PCN estimation both included k-mers as elements of the feature set. If these k-mer features were not included in the model which had maximal accuracy in testing, what is the reasoning for including them in them in two models (or at least the one which intends to use chromosomal data)?

We thank the reviewer for this important question regarding our model design choices. While the model using protein domains, plasmid length, and chromosomal length achieved marginally higher accuracy in feature-ablation tests, our downstream PCN estimation models were designed with a different priority: maximizing biological

completeness, generalizability, and robustness across diverse and potentially noisy metagenomic datasets.

We retained *k*-mer features in these final models for two key reasons:

(1) Complementary biological signal: Protein domains identify which replication and control machinery is present. K-mers, however, capture fine-grained, sequence-level information—particularly from non-coding regions like replication origins and promoter elements—that dictates how that machinery is regulated. This regulatory layer can vary significantly even among plasmids sharing identical core protein domains. Thus, *k*-mers provide information in addition to domains.

(2) Robustness to annotation gaps: In our training set, domain annotations are largely complete. In the real-world applications for which these models are intended, annotations can be incomplete, especially when analyzing novel or poorly annotated plasmids from metagenomes. K-mer features are annotation-agnostic; they are derived directly from the raw sequence and remain available even for plasmids with atypical architecture or missed domain calls. Including them ensures the model does not fail when faced with such data gaps, significantly enhancing its practical utility and reliability.

In essence, we accepted an almost negligible reduction in peak theoretical performance on a clean benchmark (as the inclusion of *k*-mers did not meaningfully degrade accuracy) to gain robustness and biological insight for applied use. The *k*-mer-inclusive model is therefore the more versatile and dependable tool for ecological inference.

We have clarified this rationale in the revised Methods section (lines 498-509) to underscore that our model selection was driven by principles of biological completeness and application robustness, not solely by cross-validation metrics.

2. Why were k-mers selected of lengths 1,2 and 3? Were longer k-mers considered to include additional sequence context and information (including key non-coding plasmid regions such as the origin of replication) at the cost of computational complexity? Would a simpler measure of sequence composition (such as G/C%) provide similar or reduced performance in the model?

We thank the reviewer for these insightful questions, which allows us to further clarify our rationale in feature selection. We selected *k*-mers of length 1–3 as a balance between capturing informative sequence composition and maintaining model robustness and computational efficiency.

In response to the reviewer's questions, we systematically evaluated longer *k*-mers and found that extending the feature set beyond $k = 3$ did not improve performance. Models

using k = 1–3 achieved stable accuracy ($R^2 = 0.738 \pm 0.013$), whereas inclusion of longer k-mers led to a small but consistent decline in performance, likely due to the rapidly expanding and increasingly sparse feature space. Short k-mers effectively avoid overfitting and unnecessary computational cost.

Furthermore, we directly compared k-mers to a simpler alternative, GC content. When used in combination with other features, GC% provided similar performance gain; however, in a direct comparison where each was used alone, k-mers substantially outperformed GC%. Therefore, we retained the k-mer representation because it offers greater interpretability, allowing identification of specific sequence patterns associated with copy-number control rather than a single global compositional metric.

These comparative analyses are now included in the Supplementary Information (Supplementary Fig. S2D and E), and we have clarified our feature-selection rationale in the revised Methods (lines 517-525).

3. Three replicates were used for testing of feature combinations in the model. The error presented in Figure S2 and Figure 2B present notable deviation in performance between these replicate runs. Additional replicate runs in order to better define the error of performance across feature combinations would improve resolution of potential differences (or lack thereof) among the similar model performance for different feature combinations.

We thank the reviewer for this helpful suggestion. In response, we increased the number of independent replicate runs from three to ten for all feature combinations.

The expanded replication provides a more reliable estimate of performance variability and improves resolution among closely performing feature sets. While minor shifts in ranking were observed among models with very similar accuracy, no qualitative changes to our conclusions occurred: feature combinations that performed well originally remain consistently high-performing, and no previously strong models became systematically inferior.

The revised analysis clarifies that several feature sets exhibit overlapping performance within error bounds, reinforcing the robustness of our conclusions. The updated results are shown in the revised Figure 2B and Supplementary Fig. S2A and B.

4. The Louvain algorithm was selected for community detection based on the protein domain content of plasmids. The Leiden algorithm was developed for community detection and has been shown to offer improvements in community detection and assurance of connectedness among detected communities as compared to the Louvain algorithm. Citation for Leiden algorithm: Traag, V.A., Waltman, L. & van Eck, N.J.

From Louvain to Leiden: guaranteeing well connected communities. Sci Rep 9, 5233 (2019). https://doi.org/10.1038/s41598-019- 41695-z.

We thank the reviewer for this helpful suggestion. In response, we repeated the community detection analysis using the Leiden algorithm, which offers improved guarantees on community connectedness.

Applying both Louvain and Leiden to the same plasmid domain similarity network (using identical edge weights and resolution parameters) yielded highly concordant results. Both methods identified 112 communities with nearly identical modularity scores (0.8054 for Louvain; 0.8055 for Leiden). Community assignments showed 99.5% average similarity, showing perfect correspondence between algorithms (Supplementary Fig. S9C). This indicates that the inferred community structure is stable and not sensitive to the choice of algorithm.

Because the biological interpretation and conclusions are unchanged, we retained the Louvain-based results in the main text for consistency with the original analysis and prior work, and we now note this robustness check in the revised Methods (lines 559–563).

5. The provided codebase contains code relating to recreation of the model and provides the model as files to be loaded for downstream use. However, the example which is provided in the repository for user testing of the model is 4A/Clinical_isolates-PCN_predictions.ipynb. This file relies on Figure_4_data.csv in order to test the model's performance on data from the clinical isolates in the study. This file does not appear to contain the columns relating to the necessary feature datapoints to run this example file (does not include the k-mers, domain presence and absence, etc.). Inclusion of an example data file for end users (which is what the github documentation implies) would be beneficial for users interested in using the provided model and code. Additionally, an environment file for installation of necessary packages or enumeration of necessary installations in the repository's readme would improve the usefulness of the provided code.

We thank the reviewer for this valuable suggestion.We have now updated the Figure_4A/Figure_4_data.zip file to include all required features (k-mers, domain presence/absence, and length features) for end users to test the model. Additionally, we have added requirements.txt and environment.yml files for package installation, along with detailed installation instructions in the README.

Minor comments:

1. Line 30: Please define the term ARG (presumably antibiotic resistance genes) at its first use.

We thank the reviewer for this comment. We have now defined ARG (antibiotic resistance gene) at its first use in the Abstract.

2. Line 25: Listing the specific number of plasmids used in model training may be preferable to “>10,000”.

Thanks for this comment. As suggested, we have revised the manuscript to specify the exact number of plasmids used in model training (11,051) instead of the approximate “>10,000”.

3. Line 388-394: Does inclusion of additional domains (through relaxation of statistical filtration) affect model performance?

We thank the reviewer for raising this important point. We explicitly evaluated the effect of progressively relaxing the statistical filtration by incrementally including domains ranked by significance. As shown in the learning curve (Supplementary Fig. S9B), test R^2 plateaus at ~0.71 around our threshold of 1,288 domains and remains flat when expanding to 12,00+ domains, demonstrating that significance-based filtering captures all predictive information while excluding non-informative features. We have revised text lines 461-463 for further clarification regarding domains selection criteria.

4. Line 408-411: Several alternatives to the selected random forest model were stated, however no performance statistics or citations reinforcing the optimality of this selection were offered.

We thank the reviewer for pointing this out. To address this concern, we performed a systematic comparison of the Random Forest model with several alternative approaches, including linear regression and XGBoost.

Models performances were evaluated using R^2 across10 independent train-test splits (80/20 split ratio) with different random seeds.. As shown in the newly added Supplementary Fig. S2C, Random Forest consistently achieved the highest R^2 with lower variance across replicate runs compared to the other models.

These results support our choice of Random Forest as a robust and well-suited model for this task, consistent with prior studies demonstrating its effectiveness for nonlinear[4,5], high-dimensional biological prediction problems. We have revised the text in lines 489 to 492 explicitly reference these comparative results.

5. Inclusion of additional details of the pre-evaluation process would improve methodological clarity.

We thank the reviewer for this helpful suggestion. To improve methodological clarity, we have expanded the description of the pre-evaluation process in the Methods section (Lines 483-525). The revised text now explicitly describes the data-splitting strategy, the use of multiple independent train–test replicates, the pre-evaluation benchmarking of alternative models and feature combinations, and the criteria used to select the final modeling framework prior to formal performance reporting. These additions clarify how modeling decisions were made and reinforce the robustness of the reported results.

6. Line 411-414: Optimal model hyperparameters are clearly calculated in the provided code and the process is documented. Were these optimal parameters for the provided models stated in the manuscript or supplementary material?

We thank the reviewer for the careful review. We have added the optimal hyperparameters to lines 493-497 of the revised manuscript.

7. Line 464: There is an extra space in “1, 288”.

We thank the reviewer for catching this typo. The extra space has now been removed.

8. Line 521: There appears to be a missing citation for Prokka.

Citation: Torsten Seemann, Prokka: rapid prokaryotic genome annotation, Bioinformatics, Volume 30, Issue 14, July 2014, Pages 2068–2069, https://doi.org/10.1093/bioinformatics/btu153

We thank the reviewer for catching this omission. The Prokka citation has now been added.

9. Some references do not contain proper usage of italics for genus and species names (such as Yersinia in reference 7).

Thanks. We have corrected the formatting of all genus and species names in the reference list to ensure proper italicization, including the one the reviewer mentioned.

10. This does not necessarily merit a change, but the order of panels in Figure 1, specifically with Figure 1G being placed in the upper right corner, is somewhat visually confusing.

Thanks. We have rearranged the panels in Figure 1 to follow a more logical and visually intuitive layout.

11. Figure 3D: Addition of the size of each community to the figure would improve interpretation.

Thanks. We have updated Figure 3D to include the size of each community as suggested.

12. Similar to Figure 1, as this does not necessarily merit change, but the placement of panel I above panel H is somewhat visually confusing.

Thanks. We have reordered panels in figure 5 to improve visual flow and clarity.

Reviewer #3 (Remarks on code availability):

The code provided includes both the calculated machine learning model and the code necessary to generate the model and analysis necessary for generation of manuscript figures. A README is included detailing the contents of the repository. Several concerns regarding the inclusion of example data and package installation instructions are listed in the comments to the author, however the provided code appears to be sufficient for recreation of the presented analysis, and the provided model is available for use for the community.

We thank the reviewer for the positive feedback. We have updated the example data file (Figure_4A/Figure_4_data.zip) to include all necessary features and added package installation files (requirements.txt and environment.yml) with installation instructions in the README as suggested.

Reference

1 Maddamsetti, R. *et al.* Scaling laws of bacterial and archaeal plasmids. *Nature Communications* **16**, 6023 (2025).

2 Ramiro-Martínez, P., de Quinto, I., Lanza, V. F., Gama, J. A. & Rodríguez-Beltrán, J. Universal rules govern plasmid copy number. *Nature Communications* **16**, 6022 (2025).

3 Ahlmann-Eltze, C. & Huber, W. Comparison of transformations for single-cell RNA-seq data. *Nature Methods* **20**, 665-672 (2023). https://doi.org/10.1038/s41592-023-01814-1

4 Zhang, Y. *et al.* Predicting functions of uncharacterized gene products from microbial communities. *Nature Biotechnology* (2025). https://doi.org/10.1038/s41587-025-02813-7

5 Lund, D. *et al.* Genetic compatibility and ecological connectivity drive the dissemination of antibiotic resistance genes. *Nature Communications* **16**, 2595 (2025). https://doi.org/10.1038/s41467-025-57825-3

Point-to-point response to reviewer's comments:

Reviewer #1 (Remarks to the Author):

Point-to-point response to authors:

I thank the authors for their detailed responses and for the substantial effort in revising the manuscript. The addition of the sensitivity analysis for large plasmids and the CQV calculations certainly strengthen the technical rigor of the work. I also appreciate the authors' candid acknowledgment that the primary aim of this study is methodological: to establish a predictive baseline for plasmid copy number (PCN) in the absence of host information.

We thank the reviewer for the careful and thorough evaluation of our revised manuscript, and for acknowledging the technical improvements made in the previous revision. We have carefully considered all remaining concerns and have addressed them as detailed below. We believe the revised manuscript now more clearly distinguishes between statistical predictive accuracy and biological insight, and appropriately frames our ecological findings as hypothesis-generating rather than conclusive.

However, while the technical execution remains commendable, the biological and ecological contextualization is still insufficient. The core conceptual limitations have not been fully resolved, and the manuscript continues to conflate statistical predictive accuracy with genuine biological insight. For the manuscript to be suitable for publication, the following discrepancies must be addressed:

The Fundamental Problem - Host Taxonomy is Not Host Physiology.
The authors argue that host-dependent variation is not the primary source of PCN variance, citing that the inclusion of host genus yielded a modest predictive improvement and that 88.7% of replicon types showed no significant PCN variation across genera. However, taxonomic classification at the genus level is fundamentally inadequate to capture host physiology. In situ PCN is dynamically shaped by strain-level variations, metabolic states, and environmental stress—factors that are completely invisible at the broad genus level. Dismissing the host's effect based on genus-level averages does not resolve the reality that PCN is an emergent property of host-plasmid-environment interactions. The manuscript must explicitly acknowledge that the apparent "host-independence" in this model is largely an artifact of coarse taxonomic resolution, rather than a proven biological reality.

We thank the reviewer for this important and insightful clarification. We agree that host taxonomy at the genus level is an imperfect proxy for host physiology, and that plasmid copy number is ultimately shaped by dynamic host–plasmid–environment interactions, including strain-level variation, metabolic state, and environmental conditions that are not captured in our dataset.

Our intention was not to dismiss host effects, but rather to assess the extent to which coarse-grained, available host information contributes to explaining PCN variance at large scale. We agree with the reviewer that the limited predictive gain from genus-level features likely reflects the restricted resolution of taxonomy as a proxy for physiology, rather than the absence of host influence per se.

At the same time, our results indicate that plasmid-encoded features alone capture a substantial fraction of the predictable variance in PCN. We interpret this as evidence that plasmids encode regulatory mechanisms that are relatively robust across broad host contexts, while finer-scale variation—such as strain-specific physiology and environmental conditions—remains unresolved in current large-scale datasets.

We have expanded the Discussion (lines 402-407) to explicitly acknowledge (i) the limitations of genus-level taxonomy, (ii) the likely contribution of unobserved host physiological variables, and (iii) the need for future work integrating strain-resolved genomics and environmental metadata to fully resolve PCN dynamics. This revised framing emphasizes that our model captures global, cross-context regularities, while recognizing that higher-resolution host information will be essential to explain residual variation.

Predictive Accuracy vs. Biological Relevance of Replicon Families.
The authors successfully demonstrate that their integrated domain-centric model statistically outperforms a replicon-only baseline, capturing intra-family variation (via the CQV analysis). However, from an ecological standpoint, the manuscript fails to demonstrate that predicting an exact numerical PCN provides a substantial biological advantage over simply identifying the plasmid family. Plasmid replicon families inherently dictate the overarching replication strategy (i.e., broad low- vs. high-copy categories), which is largely sufficient to draw the broad ecological patterns presented in this study. The incremental precision provided by the machine-learning model over this categorical baseline does not translate into transformative ecological insight. Furthermore, without host physiological data, it is impossible to definitively conclude whether this precise intra-family variance is truly driven by secondary molecular features or is merely a confounding artifact of host strain and physiological differences within the dataset. The manuscript must critically address the limited biological utility of this numerical refinement compared to replicon classification.

We thank the reviewer for this thoughtful comment and for highlighting the important distinction between statistical improvement and biological relevance. We agree that replicon family classification captures the broad replication strategies of plasmids and is sufficient to distinguish major categories such as low- and high-copy systems. Our goal is not to replace this well-established framework, but to extend it by resolving the substantial quantitative variation that exists within these categories.

Specifically, while family-level classification provides a coarse-grained view, many ecological and functional outcomes—such as gene dosage, expression levels, and antibiotic resistance amplification—depend on continuous differences in copy number rather than categorical labels. For example, variation within high-copy plasmids (e.g., 20 vs. 200 copies) can result in order-of-magnitude differences in gene dosage, which cannot be captured by family assignment alone. Our model enables this quantitative resolution, allowing more precise estimation of ecological patterns such as ARG dosage distributions and environment-specific shifts in plasmid burden.

We agree with the reviewer that, in the absence of high-resolution host physiological data, some intra-family variation may reflect unresolved host effects. We acknowledged this point in the updated manuscript (lines 411-413). Importantly, however, our results show that plasmid-encoded molecular features systematically explain a substantial fraction of this variation, suggesting that the observed differences are not solely attributable to host confounding.

We have expanded the Discussion (lines 423-428) to clarify the contributions of our framework: replicon families define the broad replication regime, while quantitative PCN prediction provides the resolution necessary to link plasmid biology to ecological and functional outcomes. This distinction better reflects the biological utility of our approach and its role in advancing from categorical to quantitative understanding of plasmid ecology.

Superficial Ecological Claims in the Abstract.
I appreciate that the authors have appropriately re-framed the ecological analysis in the revised Discussion (lines 427-434), accurately defining these findings as "hypothesis-generating" and acknowledging that current limitations in host taxonomic resolution constrain definitive ecological interpretation. However, this cautious and necessary framing is entirely absent from the Abstract. The Abstract continues to claim that the study uncovers "niche specific taxonomic PCN hotspots and ecological adaptations" (lines 28-30) and provides "critical insights into plasmid ecology." To advance to publication, these strong ecological claims in the Abstract and Introduction must be explicitly toned down to align with the methodological nature of the study and the more realistic, constrained scope established in the revised Discussion. In conclusion, the analytical scale of this study remains impressive, but to provide a meaningful biological contribution, the manuscript must ensure that its introductory and concluding claims are strictly constrained by its methodological limitations.

We thank the reviewer for this observation. We have revised the Abstract accordingly. Specifically, “the first comprehensive analysis” has been replaced with “a large-scale analysis”, and “uncovering niche specific taxonomic PCN hotspots and ecological adaptations” has been revised to “revealing putative niche-specific PCN patterns and hypothesis-generating ecological trends”. Additionally, “critical insights” has been

replaced with "valuable insights". In Introduction, we have replaced "uncovering ecosystem specific PCN trends" with "revealing putative ecosystem specific PCN trends" These changes ensure the Abstract and Introduction are consistent with the cautious framing already present in the Discussion

Reviewer #2 (Remarks to the Author):

I am happy with the revision.

We thank the reviewer for the positive assessment of the revised manuscript.

Reviewer #3 (Remarks to the Author):

The manuscript "Integrating theory and machine learning to reveal determinants of plasmid copy number" and the adjustments made to the original submission of the manuscript have resulted in significant improvements to the quality of the work. Previous concerns raised regarding the manuscript were widely addressed, including: clarification of the methodology regarding feature selection, improvements to robustness of testing framework for feature selection and model evaluation (number of replicates for feature selection, etc.), and availability of data for execution of code provided with the manuscript. Improvements to the methodology described for the machine learning framework bolster the results described in the paper. Improvements to wording based on reviewer comments have also improved the clarity of the results. Overall, this manuscript is of high quality and presents meaningful research in the field of plasmid copy number prediction and evaluation which should be of use to the community. Several minor comments are listed below.

We thank the reviewer for the positive assessment and constructive minor comments. We have addressed all comments as detailed below.

Minor Comments:
Line 546: Citation number (58 in the current manuscript) for use of scikit-learn's implementation of cosine similarity is missing.

We thank the reviewer for pointing this out. The citation for scikit-learn has been added.

Lines 571-575: Presumably default values for confidence thresholds and alignment coverage were used for MOB-typer. It would be useful to state this in the methods section.

We thank the reviewer for this suggestion. We have added the following statement to the Methods section (lines 590-591): MOB-typer was run with default parameters (minimum e-value threshold = 10^{-5}, minimum sequence identity = 80% and minimum alignment coverage = 80%).

Reviewer #3 (Remarks on code availability):

The code remains in a state which is well documented and clearly reflects the analysis described in the manuscript. Additional clarification of installation and protocol for use of code by end users and availability of the full dataset necessary for use of the code are now provided, as well. This appropriately addresses the previous concerns raised regarding the availability of code and data for the manuscript.

We thank the reviewer for the positive evaluation of the code availability and documentation.
